# The epigenetic basis of hepatocellular carcinoma – mechanisms and potential directions for biomarkers and therapeutics

**DOI:** 10.1038/s41416-025-02969-8

**Published:** 2025-03-08

**Authors:** Hong-Yi Lin, Ah-Jung Jeon, Kaina Chen, Chang Jie Mick Lee, Lingyan Wu, Shay-Lee Chong, Chukwuemeka George Anene-Nzelu, Roger Sik-Yin Foo, Pierce Kah-Hoe Chow

**Affiliations:** 1 Department of Medicine, Yong Loo Lin School of Medicine, National University of Singapore, Singapore, Singapore; 2 Department of Research and Development, Mirxes, Singapore, Singapore; 3 Department of Gastroenterology and Hepatology, Singapore General Hospital, Singapore, Singapore; 4 Cardiovascular Research Institute, National University Heart Centre, Singapore, Singapore; 5 Program in Translational and Clinical Research in Liver Cancer, National Cancer Centre Singapore, Singapore, Singapore; 6 Faculty of Medicine, University of Montreal, Quebec, Canada; 7 Department of Cardiology, National University Heart Centre, Singapore, Singapore; 8 Department of Hepato-pancreato-biliary and Transplant Surgery, Division of Surgery and Surgical Oncology, Singapore General Hospital and National Cancer Centre Singapore, Singapore, Singapore; 9 Surgery Academic Clinical Programme, Duke-NUS Medical School, Singapore, Singapore

**Keywords:** Hepatocellular carcinoma, Cancer epigenetics

## Abstract

Hepatocellular carcinoma (HCC) is the sixth leading cancer worldwide and has complex pathogenesis due to its heterogeneity, along with poor prognoses. Diagnosis is often late as current screening methods have limited sensitivity for early HCC. Moreover, current treatment regimens for intermediate-to-advanced HCC have high resistance rates, no robust predictive biomarkers, and limited survival benefits. A deeper understanding of the molecular biology of HCC may enhance tumor characterization and targeting of key carcinogenic signatures. The epigenetic landscape of HCC includes complex hallmarks of 1) global DNA hypomethylation of oncogenes and hypermethylation of tumor suppressors; 2) histone modifications, altering chromatin accessibility to upregulate oncogene expression, and/or suppress tumor suppressor gene expression; 3) genome-wide rearrangement of chromatin loops facilitating distal enhancer-promoter oncogenic interactions; and 4) RNA regulation via translational repression by microRNAs (miRNAs) and RNA modifications. Additionally, it is useful to consider etiology-specific epigenetic aberrancies, especially in viral hepatitis and metabolic dysfunction-associated steatotic liver disease (MASLD), which are the main risk factors of HCC. This article comprehensively explores the epigenetic signatures in HCC, highlighting their potential as biomarkers and therapeutic targets. Additionally, we examine how etiology-specific epigenetic patterns and the integration of epigenetic therapies with immunotherapy could advance personalized HCC treatment strategies.

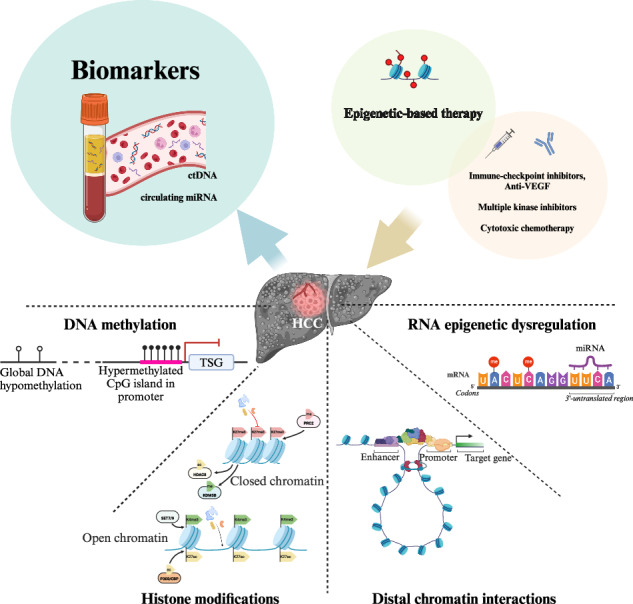

## Introduction

Hepatocellular carcinoma (HCC) is the most common type of primary liver cancer, the sixth most commonly diagnosed cancer, and the third leading cause of cancer death worldwide [1]. The incidence and mortality of HCC are projected to grow by more than 50% over the next two decades if global rates remain unchanged [1].

The early detection of HCC is presently suboptimal. The prevailing protocol for high-risk patients typically includes biannual abdominal ultrasounds supplemented by serum alpha-fetoprotein (AFP) testing [2]. However, the sensitivity for detecting early-stage HCC is only 47% with ultrasound alone, improving modestly to 63% when combined with AFP measurements [3]. This underscores the critical need for more precise diagnostic biomarkers to detect early-stage HCC reliably.

Furthermore, HCC remains a formidable clinical challenge, with a dismal 5-year survival rate of just 18%. For advanced HCC, curative options are no longer feasible. Systemic cytotoxic chemotherapy has proven largely ineffective due to the dual burden of tumor progression and underlying chronic liver disease, which predisposes patients to hepatotoxicity and myelotoxicity [4]. Recent advances in systemic therapies have offered some hope. The combination of the programmed cell death-ligand 1 (PD-L1) inhibitor atezolizumab and the vascular endothelial growth factor A (VEGFA) inhibitor bevacizumab (atezo-bev) has become a recommended first-line therapy for advanced HCC, alongside the dual checkpoint inhibitor combination of durvalumab and tremelimumab (durva-treme) [5]. These therapies marked a step forward in HCC management, providing modest improvements in survival. For instance, atezo-bev therapy achieves a 67.2% 12-month overall survival (OS) and a median progression-free survival (PFS) of 6.8 months, compared to 54.6% 12-month OS and 4.3 months PFS with the previous standard, sorafenib [6]. However, these outcomes underscore the limited efficacy of current systemic treatments, with many patients deriving only incremental benefits. For patients contraindicated for or failed atezo-bev or durva-treme, options like sorafenib, lenvatinib, or nivolumab plus ipilimumab are available, but they too fail to substantially improve survival outcomes [7]. This persistent therapeutic gap highlights the critical need for innovative approaches and a deeper understanding of HCC’s cellular and molecular biology.

The study of cancer epigenetics and its clinical applications have gained exponential popularity in recent decades. Epigenetics is the study of stable and heritable gene expression changes without any mutations to the DNA sequence [8]. The changes are primarily governed by mechanisms such as DNA methylation, histone post-translational modifications, remodeling of nucleosome positioning, and RNA interferences [9]. Epigenetic aberrations can disrupt normal cell regulation by upregulating proto-oncogenes or inactivating tumor suppressor genes, leading to uncontrolled cancer cell division and growth, chronic inflammation, and tissue fibrosis. Such disruptions often culminate in further local invasion and distal metastasis [10]. Numerous studies have established a causal relationship between epigenetic alterations and both the initiation and progression of cancer [11, 12]. This has sparked a significant debate, suggesting that cancer may not solely be a consequence of genetic mutations but could also arise from epigenetically-driven mechanisms that promote tumorigenesis [13]. Currently, epigenetic reprogramming is recognized as a critical emerging hallmark of cancer and a pivotal factor that enables the acquisition of other cancer hallmarks [14].

Studying the epigenetics of HCC opens the potential to develop early diagnostic biomarkers and avenues for targeted therapy. Using circulatory biomarkers with epigenomic alterations linked to the tumorigenesis of HCC can potentially detect HCC early and predict treatment response. In contrast to genetic mutations, epigenetic alterations are dynamic and reversible. Targeting epigenetic aberrancies and reversing them could be a potential treatment option for HCC [10].

This review aims to highlight the latest knowledge and gaps in HCC epigenetics. The potential areas of applications of epigenetics as biomarkers and therapeutics targeting HCC are also discussed.

## Epigenetic Alterations in HCC

HCC development is a multistep and chronic process, often beginning with preneoplastic fibroinflammatory changes that lead to low-grade and high-grade dysplastic hepatocytes, progressively advancing to HCC [15]. Several extrinsic factors predispose these dysplastic hepatocytes to malignant transformation, including viral hepatitis [16, 17], gut microbiome alterations [18], a hypoxic tumor microenvironment [19], and metabolic disturbances [20]. These factors often induce epigenetic alterations that are pivotal in tumor initiation. In preneoplastic lesions, key epigenetic changes include multistep hypermethylation of tumor suppressor genes such as *APC* and *RASSF1A*, alongside aberrant CpG island hypermethylation, as demonstrated by studies from Lee et al. [21] and Um et al. [22] Conversely, global hypomethylation of oncogenes, coupled with reductions in suppressive histone marks such as H3K9me3, disrupts the epigenetic equilibrium and amplifies oncogenic pathways, particularly in fibrotic livers [23].

Further insights into the extrinsic predisposing events and molecular mechanisms underlying preneoplastic HCC can be explored in studies beyond the scope of this review. The subsequent sections will focus on a detailed exploration of epigenetic aberrations, including DNA methylation, post-translational histone modifications, chromatin remodeling, and the role of non-coding RNAs in the pathogenesis of HCC [15, 24, 25].

### DNA methylation in HCC

**Fig. 1:**
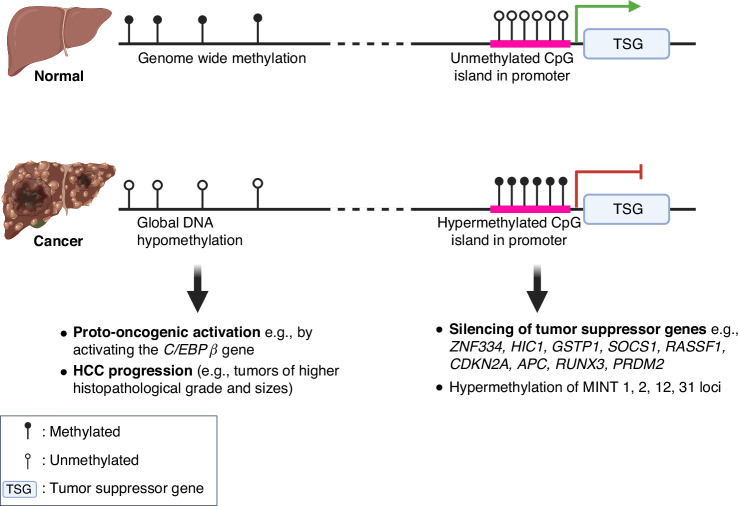
DNA methylation patterns associated with hepatocarcinogenesis. In non-cancerous hepatocytes, the genome-wide methylation and relatively unmethylated CpG island in the promoter of tumor suppressor genes suppress oncogenic expression and enable the expression of tumor suppressive pathways, respectively. In contrast, HCC is characterized by global hypomethylation, which may lead to the activation of oncogenes, e.g., the *C/EBPβ* gene, contributing to tumor progression as demonstrated by greater histopathological grades and tumor sizes. Additionally, hypermethylation at the promoter regions results in the silencing of tumor suppressor genes, e.g., *ZNF334*, *HIC1*, *GSTP1*, *SOCS1*, *RASSF1*, *CDKN2A*, *APC*, *RUNX3*, *PRDM2*. Hypermethylation at genomic regions known as methylated-in-tumor (MINT) 1, 2, 12, 31 loci are associated with hepatocarcinogenesis. These methylation changes underscore the complex regulatory mechanisms that drive the transition from normal liver function to malignant transformation.

DNA methylation is catalyzed by DNA methyltransferases (DNMTs), which typically involve the covalent addition of a methyl group to cytosine at the C5 position to form 5-methylcytosine (5mC) in CpG dinucleotides [26]. Conversely, the removal of the methyl group from cytosine is mediated through the enzymatic activity of the ten-eleven translocation (TET) family of dioxygenases (TET1, TET2, and TET3), which hydroxylate 5mC to form 5-hydroxymethylcytosine (5hmC), a precursor in the active DNA demethylation pathway [27]. In HCC, these regulatory systems are disrupted: DNMT1 and DNMT3b are overexpressed [28], while TET1 and TET2 are downregulated [29, 30]. These mechanisms, along with other complex pathways, contribute to two main patterns of DNA methylation in HCC that are strongly evidenced: (1) global hypomethylation in cancer cells relative to the normal cells and (2) focal hypermethylation of the promoters of tumor suppressor genes [31]. (Fig. 1)

#### Global hypomethylation of DNA

It has been established that approximately 80% of the CpG sequences in the genome of normal cells are methylated, whereas in tumor cells, this methylation is significantly reduced, with only about 40–60% of CpG sequences methylated [32]. This difference in methylation patterns becomes particularly relevant in the context of HCC, as the overall genomic 5-methylcytosine (5-mC) content is markedly lower in HCC compared to non-HCC liver tissues, suggesting a notable alteration in methylation patterns associated with malignancy [33]. Interestingly, this reduction in methylation is not observed when comparing normal liver to cirrhotic liver tissues [33], challenging the previously held belief that DNA hypomethylation contributes directly to the initial stages of HCC tumorigenesis [33]. Typically, other cancers show a progressive decrease in DNA methylation as they advance from normal to pre-cancerous and, finally, to cancerous stages [34]. Furthermore, the extent of genomic demethylation is particularly pronounced in HCCs with higher histopathological grades and larger tumor sizes, indicating a correlation between DNA hypomethylation and more advanced disease states [33]. These suggest that while hypomethylation may not initiate tumorigenesis, it appears to play a significant role in the progression of HCC. Correspondingly, approximately 230 hypomethylated gene promoters that are overexpressed reportedly enhanced the development and progression of HCC [35]. Significantly, hypomethylation at the enhancer regions of the *C/EBPβ* gene led to global transcriptional activation of several oncogenes in HCC [36]. Collectively, these findings underscore the critical role of DNA hypomethylation in both the progression of HCC, possibly through increasing the accessibility and activation of oncogenes in HCC.

#### DNA hypermethylation

The hypermethylation of tumor suppressor gene promoters is a significant epigenetic alteration contributing to HCC progression. This process blocks transcription factors from accessing DNA, thereby suppressing gene expression that is critical for cell growth regulation and cancer prevention. For instance, the hypermethylation of the *ZNF334* gene promoter inhibits its expression to control cell cycle and apoptosis [37]. Further investigations into HCC have shown an increase in methylation of *CDKN2A*, leading to cell cycle dysregulation [38]. Additionally, several Methylated-in-Tumor (MINT) loci – specifically MINT1, 2, 12, and 31 are found to be not only prevalent in HCC but also linked to various other cancers, suggesting a broader oncogenic role [38]. Moreover, research has identified that early stages of HCC are characterized by significant hypermethylation across a panel of tumor suppressor genes, including *HIC1*, *GSTP1*, *SOCS1*, *RASSF1*, *APC*, *RUNX3*, and *PRDM2* [39]. These findings emphasize the profound impact of DNA hypermethylation on the silencing of tumor suppressor genes in HCC, with its upstream molecular drivers in HCC remaining to be explored.

### Post-Translational Histone Modifications in HCC

**Fig. 2:**
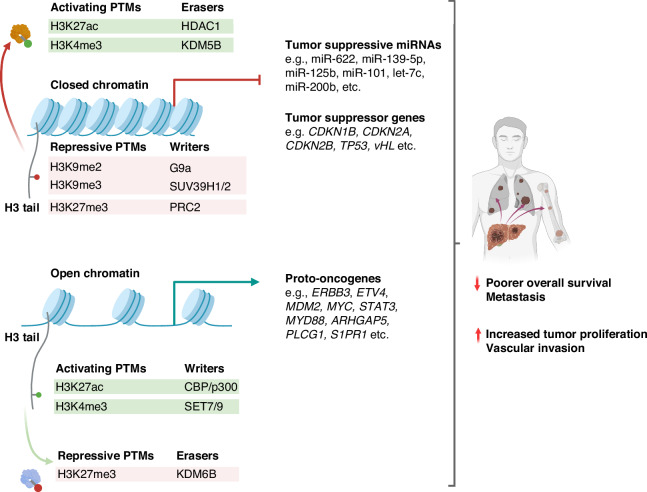
The interplay of specific histone post-translational modifications (PTMs) in HCC illustrates a complex but targeted landscape of epigenetic regulation that influences various aspects of tumor behavior and pathology. PTMs contribute to aberrant oncogenic upregulation and tumor suppressor gene repression that characterizes HCC. Activating histone PTMs (e.g., H3K27ac, H3K4me3) and erasers of repressive PTMs (e.g., KDM6B) facilitate open chromatin, leading to proto-oncogene upregulation, thereby promoting hepatocarcinogenesis. Conversely, the downregulation of tumor suppressor genes and miRNAs can be attributed to changes in chromatin accessibility induced by repressive PTMs (e.g., H3K9me2, H3K9me3, H3K27me3). Moreover, various erasers of PTM (e.g., HDAC1, KDM5B) suppress key tumor-suppressive pathways by removing activating methylation or acetylation marks. Overall, these result in increased tumor proliferation, vascular invasion, distal metastasis, and poorer overall survival.

Lysine acetylation and methylation represent quintessential histone post-translational modifications (PTMs) pivotal in gene regulation, as detailed in this review (Fig. 2). The delicate equilibrium between the addition of PTMs by writers (acetyltransferases and methyltransferases) and their removal by erasers (deacetylases and demethylases) is crucial for maintaining regulated gene expression [40].

Dysregulation of the writers and erasers of PTMs are evidenced in HCC. Acetylation of lysine residues of histone tails by histone acetyltransferases (HATs) neutralizes their positive charge, which weakens their electrostatic attraction with the negatively-charged DNA, thus increasing DNA accessibility to transcription machinery. Conversely, histone deacetylases (HDACs) restore this charge by removing acetyl groups, thus condensing chromatin structure and reducing gene expression. The specific contributions of these mechanisms to HCC development are summarized in Table 1 for acetylation [41–46] and Table 2 for deacetylation [47–54]. In histone methylation, the functional implications of histone methylation are highly variable and dependent on the type of modification and cellular context, with oncogenic implications outlined in Table 3 [42, 55–64] and 4 [65–69] for methylation and demethylation, respectively. PTMs that contribute to aberrant oncogenic upregulation and tumor suppressor gene repression that profoundly shape carcinogenesis are discussed below.

**Table 1:** Histone Acetylation in HCC.

Histone acetylated	Histone acetyltransferase	Molecular mechanisms	Potential implications in HCC	References
H3K27ac	CBP/p300	Upregulation of oncogenes e.g., *ERBB3*, *ETV4*, and *MDM2*	•HCC proliferation, invasion, and metastasis	Jin et al. [41] Liu et al. [42]
H4K8ac	PCAF	Inhibition of AKT phosphorylation Inhibition of GL1/Bcl-2/BAX pathway	•Initiation of apoptosis •Suppression of cell proliferation •Potentiate cytotoxic effects of 5-fluorouracil	Zheng et al. [43] Gai et al. [44]
H4K16ac	hMOF	Not reported	•Vascular invasion	Poté et al. [45]
H2BK120ac H3.3K18ac H4K77ac	Unknown	Not reported	•Prognostic indicator of poor survival and higher recurrence rates in patients with HCC	Chai et al. [46]

**Table 2:** Histone Deacetylation in HCC.

Histones deacetylated	Histone deacetyltransferase	Molecular mechanisms	Potential implications in HCC	References
H3K27ac	HDAC1	•Suppression of *FBP1*, *p53*, *vHL* expression •Suppression of miR-449a expression, which reduces expression of c-Met	•Portal vein invasion •Increased tumor grade and stage •Reduced overall survival •Propagation of the Warburg effect •HCC proliferation	Rikimaru et al. [47] Yang et al. [48] Kim et al. [49] Buurman et al. [50]
Not reported	HDAC2	•Suppression of miR-449a expression, which reduces expression of c-Met	•HCC proliferation	Buurman et al. [50]
Not reported	HDAC3	•Suppression of miR-449a expression, which reduces expression of c-Met •Stimulation of STAT3 signaling pathway	•HCC proliferation	Buurman et al. [50] Lu et al. [51]
H3K27ac	HDAC8	•Suppression of the expression of apoptotic proteins: BAX, BAD, BAK, caspase-3, and PARP •Activation of β-catenin signaling pathway •Inhibition of G2-M phase cell-cycle arrest •Inhibition of p53/p21 pathway	•Reduction of tumor-infiltrating CD8 + T cells •HCC proliferation •Inhibition of apoptosis •Induce insulin resistance	Yang et al. [52] Wu et al. [53] Tian et al. [54]
Not reported	HDAC9	Activation of FGFR4	•Inhibition of apoptosis	Ito et al. [71]

**Table 3:** Histone Methylation in HCC.

Histone methylated	Histone methyltransferase	Effect on gene transcription	Molecular mechanism	Potential implications in HCC	References
H3K27me1–3	PRC2	Repression	•Repression of miR-622 expression, leading to overexpression of CXCR4 •Repression of miR-139-5p, miR-125b, miR-101, let-7c, and miR-200b, resulting in overexpression of proto-oncogenes	•Increased tumor size •Vascular invasion •Reduced overall survival •Reduced recurrence-free survival after surgery	Liu et al. [55] Kusakabe et al. [56]
H3K4me3	SET7 / 9	Activation	•Upregulation of proto-oncogenes e.g., MYC, CTNNB1, STAT3, MYD88, ERBB3, ARHGAP5, PLCG1 •Overexpression of *S1PR1*, leading to upregulated AKT, STAT3, and MAPK signaling pathways •Upregulation of *CDK2* and *MMP2*	•HCC proliferation •Metastasis •Reduced overall survival	Hamamoto et al. [57] Sarris et al. [58] Zhang et al. [59] Wang et al. [60] Liu et al. [42]
H3K9me1–2	G9a	Repression	•Suppression of *RARRES3* •Suppression of *Bcl*-*G*, which inhibit p53-mediated apoptosis	•HCC proliferation •Reduced overall survival	Wei et al. [61] Nakatsuka et al. [62] Bárcena-Varela et al. [76]
H3K9me3	SUV39H1/2	Repression	•Suppression of *CDKN2A*	Expression of H3K9me3 correlate with recurrence rate and progression	Chiba et al. [77]
H4K20me1	SET8	Activation	•Upregulation of Wnt signaling pathway •Upregulation of VEGF	•HCC proliferation •Metastasis •Angiogenesis	Wu et al. [63]
H3K36me3	SMYD5	Activation	•Upregulation of Ras and Wnt signaling pathways	•HCC tumorigenesis •Reduced overall survival	Zhang et al. [64]

#### Activating Histone PTMs upregulate oncogenes in HCC by altering chromatin conformation to increase transcriptional accessibility

Chromatin structure can undergo certain PTMs to increase the accessibility of oncogenes, thereby enhancing the transcriptional accessibility of oncogenic sequences and promoting the progression of HCC. Notably, the augmentation of histone acetylation, such as the acetylation of histone H3 lysine K27 (H3K27ac) and the trimethylation of H3K4 (H3K4me3) at oncogene promoters frequently lead to their overexpression. For instance, H3K27ac, facilitated by the acetyltransferases CBP/p300 [41], has been implicated in the upregulation of oncogenes like *ERBB3*, *ETV4*, and *MDM2*, which in turn drive cell proliferation, invasion, and metastasis in HCC cell lines [42]. Moreover, overexpression of SMYD3, a methyltransferase responsible for H3K4me3, has been linked to the elevated expression of key proto-oncogenes such as *MYC*, *CTNNB1*, and *STAT3*, as well as the *CDK2* gene, all of which are associated with increased tumor size and intrahepatic metastasis in murine models of HCC [58, 60]. A comprehensive histone modification screen across HCC cell lines further corroborated these findings, showing that high levels of H3K4me3 correlated with the upregulation of proto-oncogenes *MYD88*, *ERBB3*, *ARHGAP5*, and *PLCG1* [42]. Additional human studies highlighted the role of H3K4me3-mediated *S1PR1* expression in activating downstream oncogenic signaling pathways such as PI3K-Akt, JAK-STAT, and MAPK signaling, culminating in enhanced HCC proliferation, metastasis, and reduced survival [59]. In addition, erasers such as KDM6B remove specific repressive PTMs by demethylating H3K27me3, which upregulates the *SLUG* gene. This promoted epithelial-mesenchymal transition (EMT), HCC migration, invasion, and cancer stemness, which were linked to shorter OS [69]. Significantly, a recent landmark study on *Drosophila* unveils that the transient reduction of Polycomb Repressive Complex 2 (PRC2) leads to irreversible activation of oncogenic pathways and constitutive malignant transformation despite restoring normal PRC2 levels. This finding suggests that epigenetic histone PTMs may play a crucial role in initiating self-sustaining carcinogenesis at an early stage [70]. While these dynamics have not yet been explored in HCC, investigating whether brief chromatin modifications can lead to lasting oncogenic changes holds substantial promise for developing targeted therapeutic interventions. Overall, these insights into histone modification-mediated oncogene activation elucidate critical pathways in HCC pathogenesis.

#### Closed chromatin at tumor suppressor loci in HCC results from enhanced activity of erasers, coupled with the downregulation of activating histone PTMs and the upregulation of repressive histone PTMs

HCC development is intricately linked to the dysregulation of histone modifiers that fundamentally restrict chromatin accessibility at certain tumor suppressor genes. This epigenetic reprogramming is underpinned by three key mechanisms: the upregulation of histone PTM erasers, the downregulation of activating histone PTMs, and the enrichment of repressive histone PTMs. Together, these alterations establish a transcriptionally repressive chromatin environment that silences critical regulatory tumor suppressor genes, thereby facilitating oncogenesis.

The upregulation of histone PTM erasers plays a pivotal role in HCC development by restricting accessibility to tumor suppressor genes and silencing their expression. Among these key players, histone deacetylases (HDACs) and specific histone demethylases are critical in reducing chromatin openness, thereby repressing transcription.

Histone deacetylases, particularly HDAC1, have been shown to deacetylate H3K27ac at enhancers of tumor suppressor genes such as *TP53*, *vHL*, and the *FBP1* genes, leading to their downregulation [48, 49]. This repression is associated with more aggressive HCC phenotypes, including portal vein invasion, higher tumor grade, advanced TNM staging, and reduced OS [47]. Moreover, HDAC1 contributes to the Warburg effect, enhancing HCC proliferation [48]. Furthermore, HDAC1, along with HDAC2 and HDAC3, suppresses miR-449a, a critical inhibitor of c-Met mRNA, thus fostering HCC proliferation [50]. Beyond HDAC1, other HDACs, such as HDAC8 and HDAC9, also play distinct roles in HCC. HDAC8 represses apoptotic pathways by downregulating apoptotic proteins such as BAX, BAD, BAK, caspase-3, and PARP [54], while HDAC9 induces fibroblast-growth-factor-receptor-4 (FGFR4) [71].

Similar to HDACs, specific histone demethylases such as lysine-specific histone demethylase 1 (LSD1, i.e., KDM1A) and KDM5B are implicated in HCC progression. KDM1A demethylates the activating mark H3K4me1/2, silencing tumor suppressor genes such as *PRICKLE1* and *APC* genes, contributing to HCC stemness and chemoresistance [65]. Likewise, upregulation of KDM5B in HCC demethylates the H3K4me3 promoter mark and downregulates the *CDKN2B* and *CDKN1B* genes, thereby reducing the expression of p15 and p27 proteins and leading to unchecked HCC proliferation [68].

Collectively, the upregulation of histone PTM erasers, such as HDACs and specific histone demethylases, exemplifies the multifaceted epigenetic dysregulation in HCC. By repressing tumor suppressor genes and apoptotic pathways while fostering stemness and proliferation, these enzymes create a permissive environment for tumor growth and resistance to therapy,

Furthermore, the diminished expression of activating PTM writers, which typically increases chromatin accessibility, contributes to HCC by suppressing tumor-suppressive pathways. For example, the p300/CBP-associated factor (PCAF), which acetylates histone H4 lysine K8 (H4K8ac) [72], is downregulated in HCC, enhancing the PI3K-Akt pathway, which inhibits apoptosis and promotes cell proliferation. [43] Reduced PCAF expression also contributes to HCC resistance against 5-fluorouracil through the overexpression of the GLI1/Bcl-2/BAX pathway [44, 73]. This loss of activating PTM writers exemplifies the epigenetic disruption that silences tumor suppressor networks and fosters a cellular environment conducive to tumor growth and developing therapy resistance.

Conversely, the upregulation of repressive histone methyltransferases, such as PRC2, G9a, and SUV39H1/2, reinforces tumor progression by depositing repressive histone marks. PRC2 promotes the trimethylation of histone H3 lysine K27 (H3K27me3) [74], which is correlated with increased tumor size [55], locoregional invasion [75], and poor prognosis [56]. Mechanistically, H3K27me3 represses the expression of key tumor-suppressive microRNAs such as miR-622, miR-139-5p, miR-125b, miR-101, let-7c, and miR-200b [75], thereby silencing critical anti-tumor pathways. Similarly, G9a facilitates the mono-/di-methylation of histone H3 lysine K9 (H3K9me1/2) [61], silencing tumor suppressor genes such as *RARRES3* [61] and *Bcl-G* [62] tumor suppressor genes. Also, G9a overexpression is associated with poor clinical outcomes of HCC [76]. Further exacerbating this repressive epigenetic landscape, SUV39H1/2 mediates the trimethylation of histone H3 lysine K9 (H3K9me3) [77], effectively silencing *CDKN2A*, a critical gene encoding p16 that regulates the cell cycle [78]. This led to unchecked HCC proliferation and is associated with poor prognosis [77]. Hence, these repressive histone modifications orchestrate a repressive chromatin state, transcriptionally silencing tumor suppressor genes and underpinning tumor growth and invasiveness.

#### Altered Expression of Epigenetic Readers Contributes to HCC

Epigenetic readers, key interpreters of histone modifications, play a pivotal role in regulating transcription by recruiting co-activators or co-repressors to specific genomic regions. Alterations in their expression disrupt these processes, creating a permissive epigenetic landscape that fosters tumor progression [79]. Among the emerging epigenetic readers of significance in HCC are bromodomain-containing proteins, which recognize acetylated lysine on histones and are often linked to oncogenic activation. Notably, Bromodomain-containing protein 9 (BRD9) is frequently overexpressed in HCC [80], which reportedly upregulates the Wnt/β-catenin [81] and TUFT1/AKT [82] pathways, enhancing tumor aggressiveness and correlating with poorer patient prognosis. Similarly, BRD4, another bromodomain reader, drives the transcriptional activity of NF-κB [83], a critical regulator of inflammation and tumor proliferation, further linking its upregulation to HCC progression and adverse clinical outcomes [84]. The dysregulation of these readers not only underscores their central role in epigenetic control but also positions them as promising therapeutic targets to disrupt oncogenic transcriptional programs and improve HCC treatment strategies.

### Spatial Organization of DNA and Distal Chromatin Interactions in HCC

**Fig. 3:**
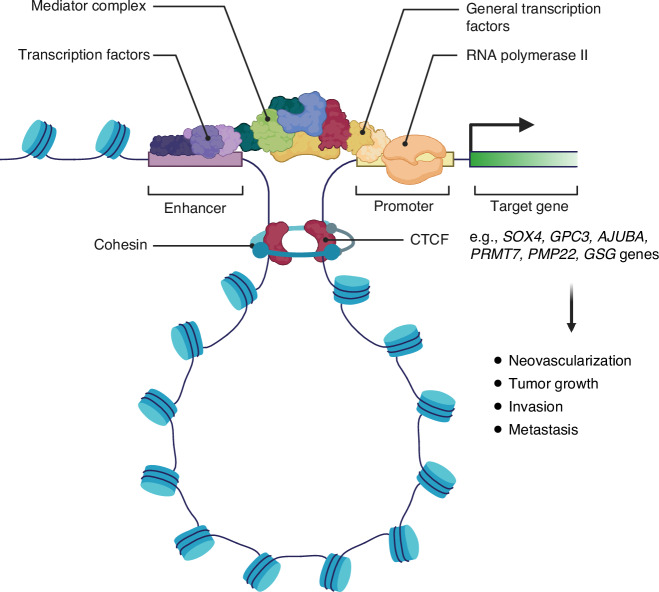
Transcriptional activation of proto-oncogenes from distal promoter-enhancer interaction. In some cases of HCC, aberrant chromatin loop formation brings de novo distal enhancers in close proximity to the promoter of several proto-oncogenes. The chromatin loop is stabilized by the CCCTC-binding factor (CTCF) and the cohesin complex to facilitate gene transcription. These interactions are associated with increased neovascularization, tumor growth, invasion, and metastasis in HCC.

Chromatins are arranged in special three-dimensional (3D) territories within the nucleus in a non-random manner [85]. The formation of chromatin loops may enable gene promoter and distal enhancer interactions [86]. Some examples of altered chromatin architecture and aberrant distal interactions observed in HCC are summarized in Fig. 3.

Genome-wide epigenetic and chromatin profiling studies reveal that HCC is characterized by a significant rearrangement of active enhancers and chromatin loops, with increased deposition of H3K27ac at tumor-specific enhancers and a higher prevalence of chromatin looping compared to adjacent normal tissues [87]. These architectural changes facilitate the upregulation of oncogenic drivers such as *SOX4* and *GPC3* [87], with *SOX4* promoting tumor neovascularization and metastasis [88], while *GPC3* activates Wnt signaling to drive HCC growth [89].

Further studies have revealed that the chromatin conformation in HCC often exhibits increased ectopic distal interactions between super-enhancers and oncogenic promoters, increasing their expression. The mechanisms underlying super-enhancer acquisition in cancer include chromosomal translocations that reposition super-enhancer elements [90], disruptions to the structure of insulated chromatin loops [91], and the aberrant formation of transcription factor binding clusters that establish super-enhancers [92]. Tsang et al. highlighted the markedly altered super-enhancer landscape in HCC, showing gains of super-enhancers at the promoters of key oncogenes such as *SPHK1*, *MYC*, *MYCN*, *SHH*, and *YAP1*, resulting in their transcriptional upregulation [93]. Additionally, increased super-enhancer interactions have been shown to upregulate the *AJUBA* gene, activating the Akt/GSK-3β/Snail signaling pathway and promoting epithelial-mesenchymal transition (EMT), thereby driving tumor invasiveness [94]. These findings underscore the critical role of super-enhancer dynamics in reshaping the oncogenic transcriptional landscape in HCC.

Additionally, disruptions in chromatin organization, such as A/B compartment switching and alterations in topologically associating domains, have been observed. These changes can result in the overexpression of genes like *PMP22* and *GSG*, driving HCC invasion and metastasis [95].

Collectively, these findings highlight the profound impact of chromatin conformation dynamics through the interplay of increased enhancer-promoter interactions and chromatin architectural aberrations in reshaping the oncogenic transcriptional landscape of HCC.

### Epigenetic regulations by RNAs in HCC

#### Mechanisms of epigenetic regulations by RNAs

**Fig. 4:**
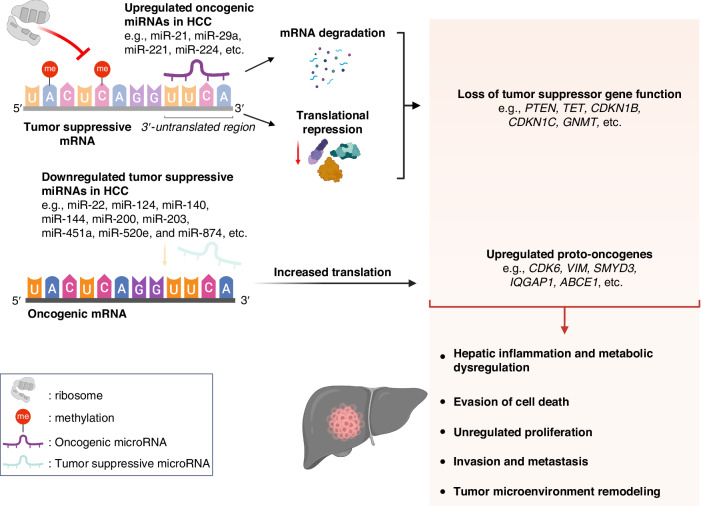
Mechanisms of epigenetic regulation by direct interference by microRNAs (miRNA) and epitranscriptomic RNA modifications. In HCC, direct miRNA interference contributes to HCC in two mechanisms. Firstly, oncogenic miRNAs degrade tumor-suppressive mRNAs and inhibit ribosomal translation. Secondly, tumor-suppressive miRNAs, which normally suppress the expression of proto-oncogenes in normal cells, are downregulated in HCC. Additionally, some epitranscriptomic changes include hypermethylation of tumor suppressor mRNA, which hinders ribosomal translation. Interestingly, hypomethylation of tumor suppressor mRNA can result in a loss in function through destabilization and degradation of mRNA. These result in increased translation of oncogenic mRNAs. Altogether, these might lead to HCC development through hepatic inflammation and metabolic dysregulation, evasion of cell death, unregulated proliferation, invasion, metastasis, and tumor microenvironment remodeling that favors hepatocarcinogenesis.

RNA regulates the epigenetic expression of DNA mainly by the following mechanisms: 1) direct interference of mRNA translation to proteins by miRNAs [96] and 2) epitranscriptomic modifications of RNA [97] (Fig. 4). The direct interference on mRNA translation by miRNAs, which bind to the 3′ untranslated region (3’-UTR) of mRNAs, hinder ribosomal translation and degrade mRNAs. Aberrant expression of miRNAs can result in carcinogenesis by hindering tumor suppressor gene expression and increasing proto-oncogene expression [98, 99]. Additionally, epitranscriptomic RNA modifications in the form of internal chemical modifications of the mRNA nucleotides without any changes to the nucleotide sequences contribute to the epigenetic regulation of gene expression by altering how mRNA is translated to proteins [100].

#### Epigenetic aberrancies caused by RNAs in HCC

miRNA dysregulations, through the upregulation of oncogenic miRNAs and the downregulation of tumor-suppressive miRNAs, have been widely reported in HCC tumorigenesis and progression.

Oncogenic miRNAs (e.g., miR-21, miR-29a, miR-221, and miR-224), which inhibit the expression of tumor suppressors, were reported to be elevated HCC. Here, Cao et al. reported that miR-21 suppressed the expression of *PTEN*, *PTENp1*, and *TET* genes in HCC cell lines [101]. Additionally, Fornari et al. studied miR-221, which suppressed the expression of *CDKN1B*/*p27* and *CDKN1C*/*p57* and, in turn, inhibited HCC cell cycle regulators in vitro [102]. Furthermore, Hung et al. investigated miR-224 in HCC cells and discovered that it inhibited the expression of *GNMT*, resulting in hepatic inflammation and metabolism disruption [103]. Finally, Chen et al. found that miR-29a silenced the *TET-SOCS1-MMP9* axis in vivo and in vitro. This reduced 5-hydroxymethylcytosine levels, which correlated with higher levels of metastasis, shorter OS, and poorer recurrence-free survival in HCC patients [104]. Altogether, these oncogenic miRNAs led to increased HCC tumor proliferation, growth, metastasis, and poor clinical outcomes.

Tumor-suppressive miRNAs (e.g., miR-22, miR-124, miR-140, miR-144, miR-200, miR-203, miR-451a, miR-520e, and miR-874), which normally inhibit oncogenic expression in normal cells, were found to be downregulated in HCC. To highlight this, Zhang et al. found that reduced levels of miR-22 increased HCC cell proliferation by upregulating HDAC4 [105]. Additionally, Furuta et al. used HCC cell lines with benign liver tissues as controls and found reduced expression of miR-124 upregulated *CDK6*, *VIM*, *SMYD3*, and *IQGAP1*. Also, reduced miR-203 levels upregulated *ABCE1*. These activated pathways led to HCC growth and metastasis [106]. Moreover, Yan et al. used murine models and reported reduced miR-140-5p expression resulted in overexpression of Pin1-mediated cancer pathways, such as cyclin D1, CDK2, Akt, ERK, and NF-κB [107]. Similarly, Zhang et al. discovered that miR-520e levels are significantly lower in human HCC tissues compared to benign liver tissues. They further elaborated that reduction in miR-520e upregulated the NIK/p-ERK1/2/NF-κB proliferative pathway in vitro [108]. Also, Zhao et al. studied miRNA expression in human HCC and reported downregulation of the miR-144/miR-451a cluster, which upregulated the hepatocyte-growth-factor and macrophage-migration-inhibitory-factor. These facilitated the polarization of M1 macrophages to M2 tumor-associated macrophages, remodeling the tumor immune microenvironment that promoted HCC initiation and proliferation [109]. Another study discovered that suppression of miR-200 in human HCC tissues was associated with EMT and metastasis [110]. Finally, Zhang et al. reported that suppression of miR-874 in HCC cell lines upregulated the DOR/EGFR/ERK pathway. Suppression of miR-874 was associated with increased tumor size, grade, and vascular invasion, as well as poorer overall and recurrence-free survival [111]. The downregulation of tumor-suppressive miRNAs may contribute to HCC development through various mechanisms, including the promotion of proliferative pathways and remodeling of the tumor immune microenvironment, ultimately driving HCC growth, invasion, and poorer clinical outcomes.

Additionally, emerging evidence highlights the pivotal role of epitranscriptomic RNA modifications in the transcriptional aberrancies underlying HCC. While the broader field of RNA modifications remains to be further investigated and is extensively discussed elsewhere [97, 112, 113], key findings specific to HCC warrant attention. One notable example is *NSUN7*, which encodes the RNA methyltransferase responsible for the 5-methylcytosine (5mC) modification [114]. Recent studies have shown that *NSUN7* is silenced in HCC cell lines, and its absence has profound downstream effects. The stability of *CCDC9B* mRNA, a regulator of the MYC proto-oncogene, relies on 5mC modification [114]. When hypomethylated, *CCDC9B* mRNA destabilizes, leading to the overexpression of *MYC*, a well-established driver of oncogenesis [114]. Similarly, N6-methyladenosine (m6A) modifications, mediated by METTL enzymes like METTL3 and METTL14, suppress tumor suppressor genes such as *SOCS2* [115]. This promotes HCC proliferation in vivo and in vitro, correlating with poorer outcomes. While the epitranscriptomic machinery remains largely under-explored, their potential as therapeutic targets positions this field as an exciting frontier in HCC epigenetics.

## Crossroads Between Epigenetic Changes and Etiological Factors in HCC

Epidemiological studies previously established several significant risk factors of HCC, such as viral hepatitis, fatty liver disease, and aflatoxin exposure [116]. Viral hepatitis is presently the most prevalent risk factor for HCC, with hepatitis B virus (HBV) infection being more common in Asia and sub-Saharan Africa, while hepatitis C virus (HCV) infection is more common in the West [116]. However, the prevalence of fatty liver is rising, especially those due to metabolic dysfunction-associated steatotic liver disease (MASLD), as the global prevalence of metabolic syndrome continues to rise from poor lifestyle and dietary habits [117]. Exposure to these risk factors may induce epigenetic changes that lead to oncogenic transformation and carcinogenesis, potentially revealing further carcinogenic mechanisms that may be targetable.

Chronic infection by HBV/HCV is a well-established risk factor for HCC, notably by inducing genomic instability and recently demonstrating capabilities to introduce epigenetic aberrancies. HBV was found to be able to integrate its DNA into the *MLL* family of genes, causing loss-of-function mutations [118]. The MLL proteins are important co-activators for H3K4me3 expression, facilitating the expression of p53-mediated genes to protect against carcinogenic DNA damage [119]. Silencing of the *MLL* genes could lead to the loss of H3K4me3-mediated protective mechanisms against HCC. Additionally, the HBV X protein (HBx) was found to upregulate the expression of DNMTs that hypermethylated and silenced tumor suppressor genes such as *IGFBP-3*, *RASSF1A*, *GSTP1*, and *CDKN2B*, which could have contributed to tumorigenesis [16]. Similarly, chronic HCV infection was found to be responsible for epigenetic aberrancies that contribute to HCC. Hamdane et al. reported global H3K27ac changes in HCV-induced HCC, which modulated the expression of genes responsible for carcinogenesis, and also correlated positively with fibrosis stage and mortality risk [17]. Altogether, chronic HBV/HCV infections could induce changes to the epigenome, which may contribute to hepatocarcinogenesis, the eventual tumor phenotype, and prognostic outcomes.

MASLD is closely linked to metabolic syndrome, where abnormal metabolic states such as hyperglycemia, dyslipidemia, and hypertension drive the accumulation of fatty acids in hepatocytes, leading to chronic inflammation, oxidative stress, fibrosis, and eventually HCC [117].

Emerging evidence highlights the role of these metabolic disturbances in inducing epigenetic changes that perpetuate carcinogenesis, even after the metabolic environment returns to normal – a phenomenon known as “metabolic memory” [120–122]. For example, a study on *Oryzias latipes* demonstrated an early-life high-fat diet induced hepatic steatosis and epigenetic changes in metabolic genes [120]. Despite subsequent normalization of the diet, a subset of genomic loci linked to liver fibrosis and HCC exhibited irreversible epigenetic alterations [120].

Classic epigenetic modifications, such as DNA methylation and histone alterations, also play a significant role in MASLD-associated HCC. In MASLD patients, DNA methylation patterns progressively favored the expression of fibrogenic genes as the severity of fatty liver disease advances, emphasizing DNA methylation’s role in transitioning MASLD to HCC [123]. Moreover, another study reported an association between decreased global DNA methylation and greater disease severity in patients with MASLD and increased risk of developing HCC [124]. Additionally, in silico analyses by Herranz et al. revealed the upregulation of *SMYD2*, a histone methyltransferase known to promote HCC tumor growth, in MASLD [125]. Similarly, *CBX1*, a histone methylation reader, was found to be upregulated in MASLD, potentially contributing to carcinogenesis [125]. Further evidence from liver biopsies highlights an enrichment of H3K27ac at genes associated with metastasis, inflammatory response, and TNF-α signaling, linking histone modifications to MASLD-induced HCC progression [126].

These findings underscore the enduring nature of metabolic insults, which establish a lasting epigenetic landscape conducive to hepatocarcinogenesis. However, how metabolic signals influence the epigenetic landscape remains to be mechanistically elucidated. Early lifestyle or pharmacological interventions to prevent or mitigate metabolic dysfunction could be key strategies in breaking this epigenetic cycle, ultimately reducing the progression of MASLD and its associated risk of HCC.

Stratifying HCC by etiologies and understanding their unique molecular features are critical for crafting targeted and effective therapies. The distinct molecular characteristics of different HCC etiologies demonstrate the inefficacy of a universal treatment strategy [117]. For instance, while immune checkpoint inhibitors (ICIs) have been approved for advanced HCC, treatment responses vary significantly depending on the underlying etiology [127]. Patients with viral-induced HCC (e.g., HBV or HCV) exhibit superior overall survival rates with ICIs compared to those with non-viral HCC, such as MASLD [127]. This discrepancy may stem from the aberrant T-cell activation in MASLD, which exacerbates tissue damage and impairs immune surveillance, ultimately diminishing the efficacy of immunotherapy [127].

Similarly, focusing on etiology-specific epigenetic traits may potentially advance the development of targeted therapies. Some promising results supporting this approach are demonstrated by the successful reduction of tumor burden in murine models of MASLD-induced HCC using bromodomain-4 inhibitors [126]. At the molecular level, these inhibitors target bromodomain-containing proteins involved in histone methylation and acetylation [128], reversing aberrant epigenetic-mediated cellular processes tied to MASLD-induced HCC, which include modulation of pathways linked to epithelial-mesenchymal transition (EMT), inflammation, and the restoration of pathways involved in bile acid, fatty acid, and xenobiotic metabolism. Likewise, inhibiting protein arginine methyltransferase 5 (PRMT5) in cell cultures and biopsies from HBV-induced HCC has shown effectiveness in reducing the dimethylation of arginine 3 on histone H4 of the covalently closed circular DNA (cccDNA) minichromosome. This alteration leads to the suppression of HBV RNA transcription, thereby hindering HBV replication within the host [129]. These findings illuminate the potential for identifying and targeting specific mechanisms inherent to different HCC etiologies, which could be pivotal in enhancing personalized treatment approaches for HCC.

## Role of Epigenetics Biomarkers in HCC

Early diagnosis of HCC is critical as it can be potentially curative with surgery, ablation, or liver transplantation. Surveillance of high-risk patients presently involves liver ultrasonography and may include serum AFP [2]. Nonetheless, ultrasonography has limited sensitivity in detecting HCC [130]. The utility of AFP is also contentious, as the biological relationship between HCC and AFP is not entirely understood [131]. Moreover, AFP is a non-specific and poorly sensitive biomarker in detecting HCC [131]. Identifying more sensitive and specific biomarkers with a strong biological basis in HCC is important for reliable diagnosis of early HCC.

Additionally, there is a pressing need for minimally invasive strategies of diagnosis and monitoring of treatment response in HCC to address the limitations of traditional surgical tissue biopsies, which are often invasive, risky, and unsuitable for repeated sampling. Liquid biopsy has emerged as a transformative modality in this context, offering a reliable, less invasive alternative for early detection and real-time monitoring of treatment response in advanced HCC [132].

### DNA methylation as biomarkers for early diagnosis and prognosis in HCC

DNA methylation markers in circulating tumor DNA (ctDNA) represent a promising frontier in HCC detection, prognosis, and therapeutic monitoring. Tumor necrosis and apoptosis release ctDNA into the bloodstream, which can be analyzed from biospecimens containing cell-free DNA (cfDNA) for specific methylation patterns associated with HCC [133]. Advances in whole-genome bisulfite sequencing have enabled genome-wide detection of DNA methylation levels, identifying epigenetic changes that closely correlate with tumor biology [134].

The utility of DNA methylation markers in circulating cell-free DNA (cfDNA) is rapidly emerging as a transformative approach for HCC detection, prognosis, and therapeutic monitoring. For instance, Xu et al. identified 18 HCC-specific methylation markers that strongly correlate with plasma ctDNA, demonstrating their potential for early detection and prognostic evaluation [135]. Building on this, Deng et al. developed the No End-repair Enzymatic Methyl-seq (NEEM-seq) method combined with a neural network model, achieving unprecedented accuracy in detecting HCC-specific methylation patterns in cfDNA [136]. Similarly, Kim et al. introduced a cfDNA methylation assay targeting *RNF135* and *LDHB*. When combined with serum AFP testing, this assay significantly improved sensitivity for HCC detection compared to AFP alone [137]. Adding further depth to this field, emerging evidence now supports integrating 5hmC signatures with 5mC profiling [138] for more comprehensive early diagnosis and recurrence detection in HCC [139]. Finally, Zhu et al. provided a comprehensive review of methylated ctDNA, highlighting the potential of methylated ctDNA in augmenting liquid biopsy-based approaches to not only detect HCC and predict its prognosis but also possibly monitor treatment responses [140].

Beyond detection, methylated ctDNA markers offer insights into HCC stratification and prognosis. Guo et al. identified 20 ctDNA methylation markers with high sensitivity and specificity for early-stage HCC, providing critical prognostic information [141]. Fu et al. expanded on these findings by reviewing distinct DNA methylation profiles associated with different HCC etiologies, such as viral hepatitis B or C and non-viral causes, underscoring the potential of methylated ctDNA to refine diagnostic accuracy and inform tailored treatment approaches [142].

These advances highlight the transformative potential of ctDNA methylation markers in HCC. By enabling non-invasive, highly specific, and clinically actionable insights into HCC biology, these tools hold promise in improving early detection, risk stratification, and personalized therapy in HCC.

### miRNAs as biomarkers for diagnosis, prognosis, and monitoring treatment response in HCC

Circulating miRNAs have emerged as a potential biomarker for the early diagnosis of HCC. A panel of seven unique miRNA markers (miR-29a, miR-29c, miR-133a, miR-143, miR-145, miR-192, and miR-505) were found to have a higher accuracy than AFP at a cutoff of 20 ng/ml in the detection of early-stage HCC (area-under-the-receiver-operating characteristic-curve 0.824 [95%CI: 0.781–0.868] vs. 0.754 [95%CI: 0.702–0.806], *p* = 0.015) [143]. Furthermore, a whole miRNome profiling study comparing HCC and non-HCC patients revealed four miRNAs (miR-1972, miR-193a-5p, miR-214-3p, and miR-365a-3p) which can differentiate HCC specifically from non-HCC patients [144]. Notably, the ongoing ELEGANCE cohort study (ClinicalTrials.gov ID: *NCT04965259*) aims to validate a panel of circulating miRNA biomarkers for developing an in-vitro diagnostic kit to detect early HCC in high-risk patients. These studies highlight the potential of circulating miRNAs to surpass current diagnostic measures by providing higher accuracy in early HCC detection and greater specificity in differentiating HCC from other tumors.

Beyond early diagnosis of HCC, circulating miRNAs are increasingly recognized for their prognostic value. Certain epigenetic alterations dysregulate gene expression, leading to aggressive tumor phenotypes and generally poorer outcomes. For example, specific plasma miRNAs – including miR-410, miR-382-5p, miR-139-5p, miR-128, miR-101-3p, and miR-424-5p – are significantly correlated with critical clinical outcomes such as OS, tumor dimensions, invasion depth, multifocality, presence of viral infection, and AFP levels [144]. Further analyses across multiple databases identified 23 miRNAs capable of predicting HCC stages, with seven miRNAs (miR-550a, miR-574, miR-424, let-7i, miR-549, miR-518b, miR-512-5p) closely linked to OS [145]. Collectively, these findings suggest that miRNAs hold substantial promise for enhancing prognostic assessments in HCC.

Additionally, circulating miRNAs have garnered significant attention as non-invasive biomarkers for predicting treatment response in advanced HCC. Notably, several miRNAs have been implicated in resistance to sorafenib, a tyrosine-kinase inhibitor for advanced HCC. For instance, miR-221 promotes sorafenib resistance by inhibiting caspase-3-mediated apoptosis, thereby impairing the drug’s pro-apoptotic effects [146]. Similarly, the overexpression of miR-216a/217 contributes to resistance through the activation of the PI3K-Akt and TGF-β signaling pathways, which are key drivers of survival and proliferation in HCC [147]. Adding to this body of evidence, a recent pivotal study identified circulating miR-518d-5p as a robust predictor of poor response to sorafenib in patients with BCLC-stage C HCC and potential as a prognostic marker [148]. With further research into biomarkers measuring treatment response, clinicians may soon be able to stratify patients based on their likelihood of response to systemic treatments, enabling the optimization of therapeutic strategies and improving outcomes in this challenging disease.

## Role of Epigenetic Therapy in HCC

Effective treatment for advanced unresectable HCC requires a good understanding of the molecular heterogeneity of HCC to target the key oncogenes with systemic therapies (Table 4). HCC is a heterogeneous tumor that forms on a background of various etiologies of chronic liver diseases, such as viral hepatitis, metabolic dysfunction, and excessive alcohol use, which drive different sets of oncogenes [149]. There are widely known genetic mutations responsible for the development of HCC, such as the *CTNNB1* gene mutation [150], *TP53* gene mutation [151], and the overexpression of the *TERT* gene [152]. However, no effective pharmaceutical agents exist to target these genetic mutations, leading to recent advocacies for a combinatory approach in targeting various molecular pathways implicated in HCC [153].

**Table 4:** Histone Demethylation in HCC.

Histones demethylated	Histone demethylase	Molecular mechanisms	Potential implications in HCC	References
H3K4me2/3	KDM1A	•Silence *Prickle1* and *APC*, which activates β-catenin pathway •Stabilize HIF-1α, which increased the expression of GLUT1 and glycolytic enzymes •Silence PGC-1α, which deactivated mitochontrial respiration •Demthylation of p65, which increases p65 stability, and increases activation of NF‐κB •Silence *GADD45B*, suppressing apoptosis and enhancing tumor survival	•HCC stemness •Chemoresistance and resistance to sorafenib •Increase glucose uptake and aerobic glycolysis	Lei et al. [65] Sakamoto et al. [66] Hu et al. [67] Sang et el. [182]
H3K4me3	KDM5B	Downregulate p15 and p27, which drove the cell cycle at the G1/S-phase	•HCC proliferation	Wang et al. [68]
H3K27me3	KDM6B	Activation of SLUG gene expression	•HCC epithelial-mesenchymal transition •HCC migration, invasion •HCC stemness •Shorter overall survival	Tang et al. [69]

Epigenetic aberrations are reversible, unlike genetic mutations. As such, epigenetic-based drugs (epidrugs) targeting epigenetic marks responsible for altering genetic expressions leading to carcinogenesis have garnered vast interest. DNMT and HDAC inhibitors were the epidrugs that were the most widely studied and the first few to be approved by the US Food and Drug Administration (FDA) for treating hematological malignancies [154–156]. These drugs were also studied in HCC in clinical trials, which will be discussed subsequently [157–159]. Other potential epidrugs to be discussed include histone methyltransferase (HMT) inhibitors, curaxins, and RNA-based therapies (Table 5). Finally, the emerging combinatorial therapy of epigenetic and immunotherapies is also discussed.

**Table 5:** List of Epidrugs, Mechanisms, and the Potential Implications in HCC.

Class/Target	Drugs	Mechanisms	Potential implications in HCC	References
**DNMT inhibitors**	**First generation**			
	Azacytidine	Chemical analogue of cytidine which inhibits DNA methyltransferase	•Reduce tumorigenicity •Potentiate effects of sorafenib	Gailhouste et al. [162]
	Decitabine	Chemical analogue of cytidine which inhibits DNA methyltransferase	•Improve progression-free and overall survival	Mei et al. [157]
	**Second generation**			
	Guadecitabine (SGI-110)	Chemical analogue of cytidine which inhibits DNA methyltransferase Inhibition of EZH2, which reduced H3K27me3 levels, leading to euchromatin	•Potentiates oxaliplatin in inhibiting HCC proliferation •Upregulate tumor suppressor genes	Kuang et al. [163]
**HDAC inhibitors**	**Hydroxamates**			
	Resminostat	•Activation of mitochondrial permeability transition pore pathway •Suppression of expression of CD44, epithelial, and mesenchymal genes •Downregulation of MEK/ERK signaling	•Induction of apoptosis •Potentiates cytotoxic effects of sorafenib by suppressing platelet-mediated HCC tumorigenesis and metastasis.	Bitzer et al. [158] Streubel et al. [167]
	Panobinostat	Upregulation of the p21 protein Induce cell-cycle arrest at the G1-phase Activation of the caspase-12 pathway	•Inhibit HCC proliferation •Inhibition of angiogenesis •Induction of apoptosis •Potentiate cytotoxic effects of sorafenib	Di Fazio et al. [168] Lachenmayer et al. [169]
	SAHA	Activation of the Notch and Raf pathways	•Induction of apoptosis •Potentiate cytotoxic effects of sorafenib	Kunnimalaiyaan et al. [170] Yuan et al. [171]
	**Short-chain fatty acids**			
	Valproic acid	•Downregulation of Jagged-2-mediated Notch-1 signaling •Activation of caspase-3	•Induce apoptosis •Overcome drug-resistance against sorafenib •Prolong overall survival in advanced HCC with manageable toxicities when given with hydralazine, gemcitabine and cisplatin, followed by doxorubicin and dacarbazine	Machado et al. [172] Liu et al. [173] Liu et al. [159]
	**Benzamides**			
	Chidamide	Upregulation of p21 mRNA expression	•Induction of apoptosis	Wang et al. [174]
	**Cyclic peptides**			
	Romidepsin	•Induction of G2/M cell cycle arrest via the Erk/cdc25C/cdc2/cyclin-B pathway •Activation of JNK/c-Jun/caspase3 pathway	•Induction of apoptosis •Reduction of HCC tumor size	Sun et al. [175]
**HMT inhibitors**	UNC0379	•Inhibition of HMT SET8 and H4K20 methylation •Inhibit the Wnt/β-catenin, promote p53 signaling pathway	•Reduce HCC proliferation •Sensitize HCC to docetaxel	Wu et al. [63]
	UNC0638	•Inhibition of G9a-mediated H3K9 methylation •Upregulation of *Bcl-G* expression to activate p53	•Induce HCC apoptosis •Reduce HCC tumorigenesis	Nakatsuka et al. [62]
	UNC1999	•Inhibition of EZH1/2-mediated H3K27 methylation	•Synergizes with sorafenib to inhibit HCC growth	Kusakabe et al. [56]
	GSK343	•Inhibition of EZH1/2-mediated H3K27 methylation	•Induction of autophagic cytotoxicity •Enhance the sensitivity of HCC cells to sorafenib	Liu et al. [181]
	Tazemetostat	•EZH2 inhibition •Inhibition of CD13 and β-catenin	•Reduce HCC development and progression	Amin et al. [178]
	Pinometostat	•DOT1L inhibition, which inhibits H3K79 •Upregulation of E-cadherin expression •Downregulation of *Snai1* and vimentin	•Attenuate the invasive epithelial-mesenchymal transition of HCC	Yang et al. [180]
	CM-272	•Inhibition of G9a-mediated H3K9 methylation •DNMT1 inhibitor •Restoration of FBP1 expression,	•Disrupts HCC’s metabolic adaptation to hypoxia, and reduces proliferation	Bárcena-Varela et al. [76]
**KDM inhibitors**	ZY0511	•Inhibition of KDM1A-mediated H3K4me1/2 demethylation	•Upregulation of apoptosis and reducing tumor survival	Sang et al. [182]
	ML324	•Inhibition of KDM4-mediated H3K4me1/2 demethylation •Activation of the ATF3-CHOP-DR5 pathway •Increase the expression of BIM	•Induce HCC apoptosis	Kim et al. [184]
**Curaxins**	CBL0137	•Inhibition of enhancer-promoter interaction of oncogenes •Inhibition of FACT, resulting in p53 activation, NF-κB suppression, and NRF2 suppression	•Reduction of tumor size •Potentiate osidative stress effects of sorafenib	Shen et al. [188]
**RNA-based therapies**	RG-101	•A potent N-acetylgalactosamine-conjugated oligonucleotide anti-miR122 with enhanced uptake by hepatocytes	•Single 2 mg/kg dose significantly reduced HCV load within 4 weeks and sustained virological response for 76 weeks •Prevent development of HCC or liver-related complications	van der Ree et al. [191]

### DNA Methyltransferase (DNMT) inhibitors in HCC

There are two generations of DNMT inhibitors. The first generation of DNMT inhibitors includes 5-azacytidine (azacytidine) and 5-azacytidine-2’-deoxycytidine (decitabine). Guadecitabine (SGI-110) is a notable example of a second-generation DNMT inhibitor.

Metabolites of DNMT inhibitors form irreversible adducts with DNMTs to inhibit their enzymatic activity, resulting in the hypomethylation of DNA [160]. In HCC, there is often hypermethylation and silencing of tumor suppressor genes. DNMT inhibitors could demethylate these genes to upregulate tumor suppressor gene expression [161].

Studies on first-generation DNMTs have shown significant potential in treating HCC. For example, pre-treatment with azacytidine before initiating sorafenib demonstrates improved cytotoxicity on HCC cells [162]. Additionally, a Phase I/II clinical trial of low-dose decitabine in patients with advanced HCC prolongs PFS and OS [157]. These findings suggest promising avenues for first-generation DNMTs in mitigating HCC tumorigenicity and improving patient outcomes.

SGI-110, a second-generation DNMT inhibitor with enhanced stability, could prevent HCC proliferation and enhance the cytotoxic effects of oxaliplatin. In studies involving HCC cell lines and mouse models, pre-treatment with SGI-110 before oxaliplatin administration significantly enhanced the suppression of HCC cell proliferation [163]. Beyond its role as a DNMT inhibitor, SGI-110 also acts as a PRC2 inhibitor, which disrupts the methylation of H3K27, thereby increasing chromatin accessibility and activating various tumor suppressor genes [164]. Consequently, SGI-110’s dual inhibition of DNMT and PRC2 facilitates the upregulation of tumor suppressor genes that are typically suppressed in HCC.

### Histone Deacetylase (HDAC) inhibitors in HCC

HDAC inhibitors are categorized into four broad classes according to their chemical structures: hydroxamates, short-chain fatty acids, benzamides, and cyclic peptides. The most widely studied class is hydroxamates [165].

Hydroxamate HDAC inhibitors (e.g., resminostat, panobinostat, suberanilohydroxamic acid [SAHA]) could inhibit proliferation, induce apoptosis, and sensitize HCC cells to sorafenib. Resminostat activates apoptosis in HCC by targeting the mitochondrial-permeability-transition-pore-pathway (mPTP) [166]. It is the only drug in this class that has been tested under a phase I/II clinical trial (SHELTER study), which found that resminostat as a single agent or combined with sorafenib is safe and efficacious [158]. The combination of resminostat and sorafenib suppresses platelet-mediated HCC tumorigenesis and metastasis by suppressing the expression of *CD44*, epithelial, and mesenchymal genes and downregulating the mitogenic MEK-ERK signaling pathway [167]. Conjointly, panobinostat inhibits HCC proliferation by cell-cycle arrest at the G1-phase from the upregulation of the p21 (CIP1/WAF1) protein, reduces angiogenesis, and induces apoptosis via the caspase-12 pathway [168]. Moreover, panobinostat potentiates the cytotoxic effects of sorafenib and decreases tumor size, which prolongs survival in mice [169]. Lastly, SAHA induces apoptosis by reducing the expression of the Notch and Raf pathways [170] and synergistically enhances the cytotoxic effects of sorafenib by the inhibition of autophagy [171]. Altogether, hydroxamate HDAC inhibitors potentially promote apoptosis and boost the cytotoxic effect of sorafenib in HCC.

Other classes of HDAC inhibitors also demonstrate the ability to inhibit the growth of HCC cells. Valproic acid, a short-chain-fatty-acid HDAC inhibitor, downregulates the Notch-1 signaling and activates caspase-3 activity, resulting in cell cycle arrest and apoptosis [172]. Additionally, valproic acid is shown to overcome drug resistance against sorafenib in HCC by downregulating the Jagged-2-mediated Notch1 signaling and the EMT-related proteins [173]. In a phase II clinical trial, a combination of valproic acid and hydralazine with gemcitabine and cisplatin, followed by doxorubicin and dacarbazine, demonstrated safety and survival benefits in advanced HCC patients [159]. Furthermore, chidamide, a benzamide HDAC inhibitor, upregulates p21 mRNA expression and induces apoptosis in HCC cells [174]. Finally, romidepsin, a cyclic peptide, activates the Erk/cdc25C/cdc2/cyclin-B and JNK/c-Jun/caspase3 pathways to trigger G2/M cell cycle arrest and apoptosis, respectively, in HCC cells. Romidepsin was also found to reduce HCC tumor size in vivo significantly [175]. Thus, various HDAC inhibitors offer a multifaceted approach against HCC development by cell-cycle arrest and inducing apoptosis. Nonetheless, human trials on these classes of HDAC inhibitors are needed to evaluate their potential safety and efficacy clinically.

There is a growing interest in the next generation of HDAC inhibitors for HCC, underscored by promising preliminary results. Among these, CXD101, a selective inhibitor targeting class I HDACs (HDAC1, HDAC2, HDAC3), has demonstrated favorable safety profiles in a Phase-I trial involving patients with advanced cancers [176].

### Histone Methyltransferase (HMT) and Lysine-Specific Histone Demethylase (KDM) Inhibitors in HCC

The inhibition of histone methyltransferases (HMTs) and lysine-specific histone demethylases (KDMs) represents a promising epigenetic therapeutic strategy in HCC. Several HMT inhibitors have already advanced to clinical trials, with tazemetostat being the most notable example [177]. This FDA-approved EZH2 inhibitor has shown efficacy in advanced epithelioid sarcoma and follicular lymphoma and has demonstrated anti-tumor activity in HCC by inhibiting *CD13* and β-catenin expression, thus curtailing tumor development and progression [178]. Similarly, pinometostat, a DOT1L inhibitor currently in Phase I trials for MLL-rearranged leukemia [179], has shown potential in HCC by upregulating E-cadherin and downregulating *Snai1* and *VIM*, thereby attenuating EMT and reducing invasiveness [180]. While promising, further studies are necessary to elucidate the full molecular mechanisms and clinical efficacy of these agents in HCC.

Preclinical studies have identified additional HMT inhibitors with specific anti-HCC activities. For instance, UNC0379 inhibits SET8 and blocks H4K20 methylation, downregulating the Wnt/β-catenin pathway while activating p53 signaling to suppress tumor proliferation and enhance chemosensitivity to docetaxel [63]. Similarly, UNC0638 inhibits G9a-mediated H3K9 methylation, leading to upregulation of the tumor suppressor *Bcl-G* and promoting p53-dependent apoptosis in liver cells with DNA damage [62]. CM-272, a dual G9a and DNMT1 inhibitor, restores *FBP1* expression, disrupts HCC’s metabolic adaptation to hypoxia, and reduces proliferation [76]. Another promising compound, UNC1999, targets both EZH1 and EZH2 to reduce H3K27me3 at the *SLFN11* promoter, thereby sensitizing HCC cells to sorafenib and enhancing anti-tumor effects [56]. Similarly, GSK343, another EZH2 inhibitor, induces autophagic cytotoxicity and improves the response of HCC cells to sorafenib [181]. Collectively, these HMT inhibitors demonstrate significant potential in restricting HCC growth, mitigating chemoresistance, and targeting key oncogenic pathways.

Conversely, KDM inhibitors leverage the induction of histone methylation to combat HCC progression. KDM1A demethylates H3K4 at the promoters of *Prickle1* and *APC*, silencing these tumor-suppressor genes and driving the β-catenin pathway, which promotes stemness and chemoresistance [65]. KDM1A also demethylates H3K4me1/2 at the promoter of *GADD45B*, suppressing its apoptotic function and enhancing tumor survival [182]. Inhibitors such as ZY0511 specifically target KDM1A activity, showing robust anti-proliferative effects in vitro and in vivo [182]. Similarly, ML324, a selective inhibitor of KDM4, which primarily demethylates histone H3 lysine 9 me2/me3 [183], induces apoptosis in HCC by activating the ATF3-CHOP-DR5 pathway and increasing the expression of BIM [184]. These KDM inhibitors not only disrupt oncogenic pathways but also restore apoptotic mechanisms, offering dual anti-tumor effects in HCC.

The emergence of HMT and KDM inhibitors underscores the therapeutic potential of targeting epigenetic regulators in HCC. These compounds, by addressing both tumor growth and chemoresistance, represent a promising direction for the development of precision therapies in HCC.

### Curaxins in HCC

Curaxins (e.g., CBL0137) is a novel anti-cancer drug class targeting the genome’s spatial organization. Unlike conventional DNA-intercalating chemotherapeutic agents, curaxins could intercalate into the DNA without notable DNA damage to increase the rigidity and modify the 3D conformation of chromatin, which inhibits enhancer-promoter communication of oncogenes [185]. Also, curaxins could inhibit the Facilitates Chromatin Transcription (FACT) complex, a histone chaperone regulating nucleosome stability and chromatin remodeling during transcription [186]. Inhibition of FACT has anti-cancer properties by activating p53 and suppressing NF-κB expression [187].

The FACT complex was found to be upregulated in HCC and contributed to HCC progression. Under oxidative stress, FACT expression is upregulated via the NRF2-KEAP1 pathway in HCC, driving NRF2 expression to increase antioxidative gene expression counteractively. Curaxin CBL0137, which is shown to be selectively effective against HCC cells in inhibiting FACT, can greatly reduce tumor size and potentiate the oxidative stress of sorafenib against HCC in vivo [188].

### RNA-based therapies in HCC

In HCC, oncogenic miRNAs are upregulated, while tumor-suppressive miRNAs are downregulated. As such, inhibiting oncogenic miRNAs and replacing tumor-suppressive miRNAs present opportunities to treat HCC. For instance, the introduction of anti-miR-221 in transgenic mice, which overexpress the oncogene miR-221, significantly reduced the quantity and volume of the HCC tumor [189]. Conversely, miR-122 is a tumor-suppressive miRNA and is often depleted in HCC. Reintroduction of miR-122 into an orthotopic HCC model reduces HCC development, invasion, metastasis, and angiogenesis [190]. Furthermore, miR-122 is an important factor for HCV proliferation, a risk factor for HCC. In a Phase-I trial, patients with HCV were randomized to receive a single 2 mg/kg dose of RG-101, a next-generation conjugated anti-miR-122 inhibitor, which demonstrated good safety and significant HCV load reduction within four weeks [191]. Herein, tumor-suppressive miRNA replacement and inhibition of oncogenic miRNA demonstrate some early promise in inhibiting the development and progression of HCC.

### Potential combination of epigenetic therapies with immunotherapies in HCC

The intersection of epigenetic therapy and immunotherapy represents a transformative approach to overcoming the limitations of immune checkpoint inhibitors (ICIs) in HCC. While ICIs targeting PD1, PD-L1, and CTLA-4 have revolutionized cancer therapy, their efficacy remains confined to a subset of patients [192], emphasizing the need for combinatorial approaches to overcome both intrinsic and acquired resistance mechanisms. Epigenetic modifications play a critical role in shaping tumor immunogenicity, regulating immune cell functionality, and modifying the tumor microenvironment (TME) [193], making them ideal candidates to complement and enhance the efficacy of ICIs in HCC. Key principles underlying this therapeutic synergy will be outlined, with additional insights available in other studies [194, 195].

#### Mechanisms of synergy between epigenetic and immunotherapy

Epigenetic therapies synergize with ICIs by modulating the immunosuppressive TME and improving immune cell infiltration in HCC. The HCC TME, dominated by tumor-associated macrophages (TAMs), myeloid-derived suppressor cells (MDSCs), and regulatory T cells (Tregs), is a major contributor to immune evasion [196]. Epigenetic modifications, including DNA methylation and histone alterations, suppress key genes essential for T-cell trafficking and activation, as well as perpetuating immune tolerance [195]. Epigenetic agents such as DNMT inhibitors, HDAC inhibitors, and HMT inhibitors counteract these effects by reactivating chemokines and cytokines, thereby enhancing immune infiltration and restoring immune sensitivity in HCC [196].

#### Potential clinical and preclinical efficacies of combinatorial epigenetic and immunotherapy

Recent studies highlight the transformative potential of combining epigenetic therapies with ICIs in HCC. For instance, HDAC8 inhibitors have demonstrated the ability to enhance the efficacy of anti-PD-L1 therapies [52]. In preclinical HCC models, selective HDAC8 inhibition reactivated chemokine production and promoted T-cell infiltration into tumors, effectively relieving T-cell exclusion. This combination resulted in sustained tumor suppression, driven by T-cell-mediated cytotoxicity, and induced robust memory T-cell responses, providing long-term immunity against tumor rechallenge [52].

Similarly, HDAC6 inhibitors have shown promise in sensitizing advanced HCC to ICIs. By reprogramming immune cells and increasing the expression of PD-L1 on tumor cells, HDAC6 inhibition enhances the antitumor activity of CD8 + T cells and amplifies immune responses, creating a more favorable tumor microenvironment [197].

Additionally, a selective class-I HDAC inhibitor, CX101, has been demonstrated to improve the treatment response of anti-PD1 inhibitors against HCC with high HDAC1/2/3 expression [198]. Mechanistically, CX101 restores the previously deficient interferon-γ/STAT1 signaling pathway, thereby promoting the recruitment of cytotoxic T-cells and increasing the expression of Gasdermin-E to induce tumor pyroptosis [198]. Building on this, a Phase-II clinical trial among HCC patients is actively being conducted to evaluate the efficacy of CXD101 in combination with the anti-PD1 inhibitor, geptanolimab, in relation to established treatments (lenvatinib or sorafenib) (ClinicalTrials.gov ID: *NCT05873244*). These strategic explorations highlight the progressive steps being taken to refine oncological treatment paradigms through combination therapies.

Emerging epigenetic targets show significant promise in modulating immune responses and hold potential candidates for combination therapies with ICIs to enhance treatment efficacy in HCC. One prospective target is KDM1A, whose inhibition amplifies T-cell-mediated cytotoxicity against HCC in vivo [199]. This effect is mediated through the upregulation of PD-L1 expression, which enhances the recruitment and activation of antitumor immune responses [199]. In addition, EZH2 inhibitors have exhibited the ability to modulate both innate and adaptive immunity. These inhibitors reprogram intra-tumoral regulatory T cells (Tregs), facilitating the recruitment of effector CD8+ and CD4 + T cells to combat HCC in vivo [200]. Beyond T-cell modulation, EZH2 inhibition also enhances the cytotoxic activity of natural killer (NK) cells by upregulating activating ligands, such as NKG2D ligands, thereby bolstering innate immune responses against the tumor [201]. Moreover, DNMT inhibitors act by upregulating endogenous retroviruses, reactivating silenced immune pathways, and enhancing the immunogenicity of tumor cells [164]. These findings highlight the potential of targeting epigenetic mechanisms, including KDM1A, EZH2, and DNA methylation, in combination with ICIs to overcome immune resistance and achieve more robust and durable antitumor responses.

#### Epigenetic biomarkers in predicting response to immunotherapy

Epigenetic biomarkers have the potential to significantly enhance the prediction of response to immunotherapy by identifying key immunomodulatory signatures that influence treatment outcomes [193]. DNA methylation-based deconvolution analysis has uncovered specific DNA methylation patterns that drive immunosuppression, even in tumors initially classified as “hot” due to higher cytotoxic T-lymphocyte (CTL) infiltration levels [202]. These suppressive signatures can hinder the efficacy of immune checkpoint blockade by promoting immune evasion.

Notably, the expression of EZH2 and DNMT1 in tumors has been shown to repress the production of T-helper 1 type chemokines, such as CXCL9 and CXCL10, which are crucial for recruiting CD8 + T cells into the TME [203]. This repression correlates with reduced infiltration of tumor-infiltrating lymphocytes and poorer clinical outcomes [203]. Moreover, EZH2-driven epigenetic modifications can directly suppress the expression of PD-L1 by increasing H3K27me3 levels at the promoters of *CD274* and *IRF1* in HCC tissues [204]. This mechanism further impairs immune recognition and contributes to immune resistance.

Given the complex interplay of factors influencing immunotherapy responses – ranging from immune checkpoint expression [205] and genetic mutations [206] to the cellular composition of the TME [207] – there is a critical need for integrative approaches to patient stratification. Combining epigenetic biomarkers with advanced predictive algorithms that incorporate multi-omics data and spatial profiling of the TME [208] could significantly improve the sensitivity and accuracy of response predictions. Additionally, integrating liquid biopsy analyses may offer a minimally invasive method to capture dynamic changes in tumor biology [209]. Such comprehensive approaches refine patient selection for immunotherapy and identify opportunities to incorporate epigenetic therapies as adjuncts, thereby enhancing treatment efficacy and overcoming resistance.

## Conclusions

This review presents the epigenetic aberrancies in HCC, notably in DNA methylation, post-translational histone modifications, alteration to the 3D conformation of the genome, and RNA regulations. We also discuss the importance of considering etiology-specific epigenetic mechanisms for more efficacious targeted treatment of HCC of different etiologies. Finally, we highlight the promise of using the epigenetic biomarkers for HCC and potentially as targets for anti-cancer therapy.

While the field of cancer epigenetics is developing rapidly, there are several limitations to its application in clinical practice. A key challenge lies in the lack of robust and validated predictive biomarkers for HCC, especially for measuring treatment response to systemic therapies in advanced stages of the disease. Although circulating biomarkers such as circulating tumor cells [210], circulating DNA [211], and extracellular vesicles [212] show promise, studies focusing specifically on epigenetic-based circulating biomarkers remain limited [213], except for some miRNAs [146–148]. This gap is particularly critical given the intimate role of epigenetic mechanisms in HCC pathogenesis and progression. Developing reliable, non-invasive epigenetic biomarkers could complement critical insights into disease monitoring and therapeutic response.

Additionally, while epidrugs targeting epigenetic modifications hold immense therapeutic potential, they are often associated with elusive and unpredictable side effect profiles. Many epidrugs exert genome-wide effects, leading to off-target consequences that are not always specific to the tumor, raising concerns about their safety [214]. Compounding this issue, the limited number of clinical studies validating the safety and efficacy of epidrugs in HCC patients further restricts their integration into routine practice. Addressing these challenges through rigorous biomarker validation and comprehensive clinical trials will be essential to fully harness the transformative potential of epigenetics in HCC diagnosis and treatment.

Nonetheless, the epigenetics of HCC remain exciting to explore as they potentially open doors to novel preventive measures and treatments. Growing evidence points to epigenetically-driven carcinogenesis, which should be detected early and targeted [11–13]. Furthermore, HCC is an aggressive malignancy with over 60% detected at advanced stages due to present surveillance inadequacies [215]. Knowledge of the epigenetic signatures of HCC could potentially be applied to complement existing diagnostics to improve the accuracy of detecting HCC, predict response to treatment, and prognosticate patients. Furthermore, epidrugs have demonstrated capabilities to work synergistically with existing anti-tumor agents against HCC and even reverse resistance against these agents [56, 63, 158, 162, 163, 169, 171, 173, 180, 181, 188]. As HCC has wide tumor microenvironmental and systemic epigenetic changes [87], epidrugs may create a more supportive cellular environment for anti-tumor agents to work effectively against HCC [216]. Additionally, in the current landscape where epidrugs have been approved by the FDA for other malignancies [154–156], epidrugs remain a promising adjunct in addition to other combination therapies for personalized treatment against HCC. With further validation in clinical cohorts, knowledge of the epigenetics of HCC can potentially be translated to develop useful clinical biomarkers and treatment targets.

## References

[1] Rumgay H, Arnold M, Ferlay J, Lesi O, Cabasag CJ, Vignat J, et al. Global burden of primary liver cancer in 2020 and predictions to 2040. J Hepatol. 2022;77:1598–606. 36208844 10.1016/j.jhep.2022.08.021 PMC9670241

[2] Heimbach JK, Kulik LM, Finn RS, Sirlin CB, Abecassis MM, Roberts LR, et al. AASLD guidelines for the treatment of hepatocellular carcinoma. Hepatology. 2018;67:358–80. 28130846 10.1002/hep.29086

[3] Tzartzeva K, Obi J, Rich NE, Parikh ND, Marrero JA, Yopp A, et al. Surveillance Imaging and Alpha Fetoprotein for Early Detection of Hepatocellular Carcinoma in Patients With Cirrhosis: A Meta-analysis. Gastroenterology. 2018;154:1706–18.e1. 29425931 10.1053/j.gastro.2018.01.064 PMC5927818

[4] Yeo W, Mok TS, Zee B, Leung TW, Lai PB, Lau WY, et al. A randomized phase III study of doxorubicin versus cisplatin/interferon alpha-2b/doxorubicin/fluorouracil (PIAF) combination chemotherapy for unresectable hepatocellular carcinoma. J Natl Cancer Inst. 2005;97:1532–8. 16234567 10.1093/jnci/dji315

[5] Gordan JD, Kennedy EB, Abou-Alfa GK, Beal E, Finn RS, Gade TP, et al. Systemic Therapy for Advanced Hepatocellular Carcinoma: ASCO Guideline Update. J Clin Oncol. 2024;42:1830–50. 38502889 10.1200/JCO.23.02745

[6] Finn RS, Qin S, Ikeda M, Galle PR, Ducreux M, Kim T-Y, et al. Atezolizumab plus Bevacizumab in Unresectable Hepatocellular Carcinoma. N. Engl J Med. 2020;382:1894–905. 32402160 10.1056/NEJMoa1915745

[7] Melero I, Yau T, Kang YK, Kim TY, Santoro A, Sangro B, et al. Nivolumab plus ipilimumab combination therapy in patients with advanced hepatocellular carcinoma previously treated with sorafenib: 5-year results from CheckMate 040. Ann Oncol. 2024;35:537–48. 38844309 10.1016/j.annonc.2024.03.005

[8] Berger SL, Kouzarides T, Shiekhattar R, Shilatifard A. An operational definition of epigenetics. Genes Dev. 2009;23:781–3. 19339683 10.1101/gad.1787609 PMC3959995

[9] Allis CD, Caparros M-L, Jenuwein T, Reinberg D. Epigenetics, Second Edition. Lachner M, editor: Cold Spring Harbor Laboratory Press; 2015.

[10] Wahid B, Ali A, Rafique S, Idrees M. New Insights into the Epigenetics of Hepatocellular Carcinoma. BioMed Res Int. 2017;2017:1609575. 28401148 10.1155/2017/1609575 PMC5376429

[11] Terekhanova NV, Karpova A, Liang WW, Strzalkowski A, Chen S, Li Y, et al. Epigenetic regulation during cancer transitions across 11 tumour types. Nature. 2023;623:432–41. 37914932 10.1038/s41586-023-06682-5 PMC10632147

[12] Flavahan WA, Gaskell E, Bernstein BE. Epigenetic plasticity and the hallmarks of cancer. Science. 2017;357:eaal2380. 10.1126/science.aal2380 PMC5940341 28729483

[13] Vicente-Dueñas C, Hauer J, Cobaleda C, Borkhardt A, Sánchez-García I. Epigenetic Priming in Cancer Initiation. Trends Cancer. 2018;4:408–17. 29860985 10.1016/j.trecan.2018.04.007

[14] Hanahan D. Hallmarks of Cancer: New Dimensions. Cancer Discov. 2022;12:31–46. 35022204 10.1158/2159-8290.CD-21-1059

[15] Thorgeirsson SS, Grisham JW. Molecular pathogenesis of human hepatocellular carcinoma. Nat Genet. 2002;31:339–46. 12149612 10.1038/ng0802-339

[16] Park IY, Sohn BH, Yu E, Suh DJ, Chung YH, Lee JH, et al. Aberrant epigenetic modifications in hepatocarcinogenesis induced by hepatitis B virus X protein. Gastroenterology. 2007;132:1476–94. 17408664 10.1053/j.gastro.2007.01.034

[17] Hamdane N, Jühling F, Crouchet E, El Saghire H, Thumann C, Oudot MA, et al. HCV-Induced Epigenetic Changes Associated With Liver Cancer Risk Persist After Sustained Virologic Response. Gastroenterology. 2019;156:2313–29.e7. 30836093 10.1053/j.gastro.2019.02.038 PMC8756817

[18] Ha S, Wong VW-S, Zhang X, Yu J. Interplay between gut microbiome, host genetic and epigenetic modifications in MASLD and MASLD-related hepatocellular carcinoma. Gut. 2025;74:141–52. 10.1136/gutjnl-2024-332398 PMC11671994 38950910

[19] Li T, Mao C, Wang X, Shi Y, Tao Y. Epigenetic crosstalk between hypoxia and tumor driven by HIF regulation. J Exp Clin Cancer Res. 2020;39:224. 33109235 10.1186/s13046-020-01733-5 PMC7592369

[20] Puszyk WM, Trinh TL, Chapple SJ, Liu C. Linking metabolism and epigenetic regulation in development of hepatocellular carcinoma. Lab Investig. 2013;93:983–90. 23917878 10.1038/labinvest.2013.94 PMC4028619

[21] Lee S, Lee HJ, Kim J-H, Lee H-S, Jang JJ, Kang GH. Aberrant CpG Island Hypermethylation Along Multistep Hepatocarcinogenesis. Am J Pathol. 2003;163:1371–8. 14507645 10.1016/S0002-9440(10)63495-5 PMC1868296

[22] Um TH, Kim H, Oh BK, Kim MS, Kim KS, Jung G, et al. Aberrant CpG island hypermethylation in dysplastic nodules and early HCC of hepatitis B virus-related human multistep hepatocarcinogenesis. J Hepatol. 2011;54:939–47. 21145824 10.1016/j.jhep.2010.08.021

[23] Chappell G, Kutanzi K, Uehara T, Tryndyak V, Hong H-H, Hoenerhoff M, et al. Genetic and epigenetic changes in fibrosis-associated hepatocarcinogenesis in mice. Int J Cancer. 2014;134:2778–88. 24242335 10.1002/ijc.28610 PMC4209252

[24] Libbrecht L, Desmet V, Roskams T. Preneoplastic lesions in human hepatocarcinogenesis. Liver Int. 2005;25:16–27. 15698394 10.1111/j.1478-3231.2005.01016.x

[25] Czauderna C, Poplawski A, O’Rourke CJ, Castven D, Pérez-Aguilar B, Becker D, et al. Epigenetic modifications precede molecular alterations and drive human hepatocarcinogenesis. JCI Insight. 2021;6:e146196. 10.1172/jci.insight.146196 PMC8492348 34375307

[26] Robertson KD. DNA methylation and human disease. Nat Rev Genet. 2005;6:597–610. 16136652 10.1038/nrg1655

[27] Bhutani N, Burns DavidM, Blau HelenM. DNA Demethylation Dynamics. Cell. 2011;146:866–72. 21925312 10.1016/j.cell.2011.08.042 PMC3236603

[28] Nakamura NagaiM, Makino A, Mitamura R. K. Expression of DNA (5-cytosin)-methyltransferases (DNMTs) in hepatocellular carcinomas. Hepatol Res. 2003;26:186–91. 12850690 10.1016/s1386-6346(03)00091-3

[29] Yang H, Liu Y, Bai F, Zhang JY, Ma SH, Liu J, et al. Tumor development is associated with decrease of TET gene expression and 5-methylcytosine hydroxylation. Oncogene. 2013;32:663–9. 22391558 10.1038/onc.2012.67 PMC3897214

[30] Wang P, Yan Y, Yu W, Zhang H. Role of ten-eleven translocation proteins and 5-hydroxymethylcytosine in hepatocellular carcinoma. Cell Prolif. 2019;52:e12626. 31033072 10.1111/cpr.12626 PMC6668972

[31] Jones PA, Laird PW. Cancer epigenetics comes of age. Nat Genet. 1999;21:163–7. 9988266 10.1038/5947

[32] Baylin SB, Jones PA. Epigenetic Determinants of Cancer. Cold Spring Harb Perspect Biol. 2016;8:a019505. 10.1101/cshperspect.a019505 PMC5008069 27194046

[33] Lin CH, Hsieh SY, Sheen IS, Lee WC, Chen TC, Shyu WC, et al. Genome-wide hypomethylation in hepatocellular carcinogenesis. Cancer Res. 2001;61:4238–43. 11358850

[34] Cravo M, Pinto R, Fidalgo P, Chaves P, Glória L, Nobre-Leitão C, et al. Global DNA hypomethylation occurs in the early stages of intestinal type gastric carcinoma. Gut. 1996;39:434–8. 8949650 10.1136/gut.39.3.434 PMC1383352

[35] Stefanska B, Huang J, Bhattacharyya B, Suderman M, Hallett M, Han Z-G, et al. Definition of the Landscape of Promoter DNA Hypomethylation in Liver Cancer. Cancer Res. 2011;71:5891–903. 21747116 10.1158/0008-5472.CAN-10-3823

[36] Xiong L, Wu F, Wu Q, Xu L, Cheung OK, Kang W, et al. Aberrant enhancer hypomethylation contributes to hepatic carcinogenesis through global transcriptional reprogramming. Nat Commun. 2019;10:335. 30659195 10.1038/s41467-018-08245-z PMC6338783

[37] Sun D, Gan X, Liu L, Yang Y, Ding D, Li W, et al. DNA hypermethylation modification promotes the development of hepatocellular carcinoma by depressing the tumor suppressor gene ZNF334. Cell Death Dis. 2022;13:446. 35534462 10.1038/s41419-022-04895-6 PMC9085879

[38] Kondo Y, Kanai Y, Sakamoto M, Mizokami M, Ueda R, Hirohashi S. Genetic Instability and Aberrant DNA Methylation in Chronic Hepatitis and Cirrhosis—A Comprehensive Study of Loss of Heterozygosity and Microsatellite Instability at 39 Loci and DNA Hypermethylation on 8 CpG Islands in Microdissected Specimens From Patients With Hepatocellular Carcinoma. Hepatology. 2000;32:970–9. 11050047 10.1053/jhep.2000.19797

[39] Nishida N, Kudo M, Nagasaka T, Ikai I, Goel A. Characteristic patterns of altered DNA methylation predict emergence of human hepatocellular carcinoma. Hepatology. 2012;56:994–1003. 22407776 10.1002/hep.25706

[40] Shen H, Laird PW. Interplay between the cancer genome and epigenome. Cell. 2013;153:38–55. 23540689 10.1016/j.cell.2013.03.008 PMC3648790

[41] Jin Q, Yu LR, Wang L, Zhang Z, Kasper LH, Lee JE, et al. Distinct roles of GCN5/PCAF-mediated H3K9ac and CBP/p300-mediated H3K18/27ac in nuclear receptor transactivation. Embo J. 2011;30:249–62. 21131905 10.1038/emboj.2010.318 PMC3025463

[42] Liu Y-X, Li Q-Z, Cao Y-N, Zhang L-Q. Identification of key genes and important histone modifications in hepatocellular carcinoma. Computational Struct Biotechnol J. 2020;18:2657–69. 10.1016/j.csbj.2020.09.013 PMC7533298 33033585

[43] Zheng X, Gai X, Ding F, Lu Z, Tu K, Yao Y, et al. Histone acetyltransferase PCAF Up-regulated cell apoptosis in hepatocellular carcinoma via acetylating histone H4 and inactivating AKT signaling. Mol Cancer. 2013;12:96. 23981651 10.1186/1476-4598-12-96 PMC3847488

[44] Gai X, Tu K, Li C, Lu Z, Roberts LR, Zheng X. Histone acetyltransferase PCAF accelerates apoptosis by repressing a GLI1/BCL2/BAX axis in hepatocellular carcinoma. Cell Death Dis. 2015;6:e1712. 25855960 10.1038/cddis.2015.76 PMC4650545

[45] Poté N, Cros J, Laouirem S, Raffenne J, Negrão M, Albuquerque M, et al. The histone acetyltransferase hMOF promotes vascular invasion in hepatocellular carcinoma. Liver Int. 2020;40:956–67. 31943753 10.1111/liv.14381

[46] Chai X, Guo J, Dong R, Yang X, Deng C, Wei C, et al. Quantitative acetylome analysis reveals histone modifications that may predict prognosis in hepatitis B-related hepatocellular carcinoma. Clin Transl Med. 2021;11:e313. 33783990 10.1002/ctm2.313 PMC7939233

[47] Rikimaru T, Taketomi A, Yamashita Y-i, Shirabe K, Hamatsu T, Shimada M, et al. Clinical Significance of Histone Deacetylase 1 Expression in Patients with Hepatocellular Carcinoma. Oncology. 2007;72:69–74. 18004079 10.1159/000111106

[48] Yang J, Jin X, Yan Y, Shao Y, Pan Y, Roberts LR, et al. Inhibiting histone deacetylases suppresses glucose metabolism and hepatocellular carcinoma growth by restoring FBP1 expression. Sci Rep. 2017;7:43864. 28262837 10.1038/srep43864 PMC5338333

[49] Kim MS, Kwon HJ, Lee YM, Baek JH, Jang J-E, Lee S-W, et al. Histone deacetylases induce angiogenesis by negative regulation of tumor suppressor genes. Nat Med. 2001;7:437–43. 11283670 10.1038/86507

[50] Buurman R, Gürlevik E, Schäffer V, Eilers M, Sandbothe M, Kreipe H, et al. Histone Deacetylases Activate Hepatocyte Growth Factor Signaling by Repressing MicroRNA-449 in Hepatocellular Carcinoma Cells. Gastroenterology. 2012;143:811–20.e15. 22641068 10.1053/j.gastro.2012.05.033

[51] Lu X-F, Cao X-Y, Zhu Y-J, Wu Z-R, Zhuang X, Shao M-Y, et al. Histone deacetylase 3 promotes liver regeneration and liver cancer cells proliferation through signal transducer and activator of transcription 3 signaling pathway. Cell Death Dis. 2018;9:398. 29540666 10.1038/s41419-018-0428-x PMC5852132

[52] Yang W, Feng Y, Zhou J, Cheung OK-W, Cao J, Wang J, et al. A selective HDAC8 inhibitor potentiates antitumor immunity and efficacy of immune checkpoint blockade in hepatocellular carcinoma. Sci Transl Med. 2021;13:eaaz6804. 33827976 10.1126/scitranslmed.aaz6804

[53] Wu J, Du C, Lv Z, Ding C, Cheng J, Xie H, et al. The Up-Regulation of Histone Deacetylase 8 Promotes Proliferation and Inhibits Apoptosis in Hepatocellular Carcinoma. Digestive Dis Sci. 2013;58:3545–53. 10.1007/s10620-013-2867-7 24077923

[54] Tian Y, Wong VW, Wong GL, Yang W, Sun H, Shen J, et al. Histone Deacetylase HDAC8 Promotes Insulin Resistance and β-Catenin Activation in NAFLD-Associated Hepatocellular Carcinoma. Cancer Res. 2015;75:4803–16. 26383163 10.1158/0008-5472.CAN-14-3786

[55] Liu H, Liu Y, Liu W, Zhang W, Xu J. EZH2-mediated loss of miR-622 determines CXCR4 activation in hepatocellular carcinoma. Nat Commun. 2015;6:8494. 26404566 10.1038/ncomms9494 PMC4598861

[56] Kusakabe Y, Chiba T, Oshima M, Koide S, Rizq O, Aoyama K, et al. EZH1/2 inhibition augments the anti-tumor effects of sorafenib in hepatocellular carcinoma. Sci Rep. 2021;11:21396. 34725436 10.1038/s41598-021-00889-0 PMC8560765

[57] Hamamoto R, Furukawa Y, Morita M, Iimura Y, Silva FP, Li M, et al. SMYD3 encodes a histone methyltransferase involved in the proliferation of cancer cells. Nat Cell Biol. 2004;6:731–40. 15235609 10.1038/ncb1151

[58] Sarris ME, Moulos P, Haroniti A, Giakountis A, Talianidis I. Smyd3 Is a Transcriptional Potentiator of Multiple Cancer-Promoting Genes and Required for Liver and Colon Cancer Development. Cancer Cell. 2016;29:354–66. 26908355 10.1016/j.ccell.2016.01.013

[59] Zhang H, Zheng Z, Zhang R, Yan Y, Peng Y, Ye H, et al. SMYD3 promotes hepatocellular carcinoma progression by methylating S1PR1 promoters. Cell Death Dis. 2021;12:731. 34301921 10.1038/s41419-021-04009-8 PMC8302584

[60] Wang Y, Xie B-h, Lin W-h, Huang Y-h, Ni J-y, Hu J, et al. Amplification of SMYD3 promotes tumorigenicity and intrahepatic metastasis of hepatocellular carcinoma via upregulation of CDK2 and MMP2. Oncogene. 2019;38:4948–61. 30842588 10.1038/s41388-019-0766-x

[61] Wei L, Chiu DK, Tsang FH, Law CT, Cheng CL, Au SL, et al. Histone methyltransferase G9a promotes liver cancer development by epigenetic silencing of tumor suppressor gene RARRES3. J Hepatol. 2017;67:758–69. 28532996 10.1016/j.jhep.2017.05.015

[62] Nakatsuka T, Tateishi K, Kato H, Fujiwara H, Yamamoto K, Kudo Y, et al. Inhibition of histone methyltransferase G9a attenuates liver cancer initiation by sensitizing DNA-damaged hepatocytes to p53-induced apoptosis. Cell Death Dis. 2021;12:99. 33468997 10.1038/s41419-020-03381-1 PMC7815717

[63] Wu J, Qiao K, Du Y, Zhang X, Cheng H, Peng L, et al. Downregulation of histone methyltransferase SET8 inhibits progression of hepatocellular carcinoma. Sci Rep. 2020;10:4490. 32161353 10.1038/s41598-020-61402-7 PMC7066161

[64] Zhang Y, Fang Y, Tang Y, Han S, Jia J, Wan X, et al. SMYD5 catalyzes histone H3 lysine 36 trimethylation at promoters. Nat Commun. 2022;13:3190. 35680905 10.1038/s41467-022-30940-1 PMC9184575

[65] Lei ZJ, Wang J, Xiao HL, Guo Y, Wang T, Li Q, et al. Lysine-specific demethylase 1 promotes the stemness and chemoresistance of Lgr5+ liver cancer initiating cells by suppressing negative regulators of β-catenin signaling. Oncogene. 2015;34:3188–98. 25893304 10.1038/onc.2015.129

[66] Sakamoto A, Hino S, Nagaoka K, Anan K, Takase R, Matsumori H, et al. Lysine Demethylase LSD1 Coordinates Glycolytic and Mitochondrial Metabolism in Hepatocellular Carcinoma Cells. Cancer Res. 2015;75:1445–56. 25649769 10.1158/0008-5472.CAN-14-1560

[67] Hu B, Xu Y, Li YC, Huang JF, Cheng JW, Guo W, et al. CD13 promotes hepatocellular carcinogenesis and sorafenib resistance by activating HDAC5-LSD1-NF-κB oncogenic signaling. Clin Transl Med. 2020;10:e233. 33377659 10.1002/ctm2.233 PMC7708822

[68] Wang D, Han S, Peng R, Jiao C, Wang X, Yang X, et al. Depletion of histone demethylase KDM5B inhibits cell proliferation of hepatocellular carcinoma by regulation of cell cycle checkpoint proteins p15 and p27. J Exp Clin Cancer Res. 2016;35:37. 26911146 10.1186/s13046-016-0311-5 PMC4766611

[69] Tang B, Qi G, Tang F, Yuan S, Wang Z, Liang X, et al. Aberrant JMJD3 Expression Upregulates Slug to Promote Migration, Invasion, and Stem Cell-Like Behaviors in Hepatocellular Carcinoma. Cancer Res. 2016;76:6520–32. 27651311 10.1158/0008-5472.CAN-15-3029

[70] Parreno V, Loubiere V, Schuettengruber B, Fritsch L, Rawal CC, Erokhin M, et al. Transient loss of Polycomb components induces an epigenetic cancer fate. Nature. 2024;629:688–96. 38658752 10.1038/s41586-024-07328-w PMC11096130

[71] Ito R, Miyanishi K, Kubo T, Hamaguchi K, Osuga T, Tanaka S, et al. Synergistic antitumor effect of histone deacetylase class IIa inhibitor with lenvatinib in hepatocellular carcinoma. Hepatol Int. 2023;17:735–44. 36738397 10.1007/s12072-023-10484-2

[72] Schiltz RL, Mizzen CA, Vassilev A, Cook RG, Allis CD, Nakatani Y. Overlapping but Distinct Patterns of Histone Acetylation by the Human Coactivators p300 and PCAF within Nucleosomal Substrates. J Biol Chem. 1999;274:1189–92. 9880483 10.1074/jbc.274.3.1189

[73] Violette S, Poulain L, Dussaulx E, Pepin D, Faussat AM, Chambaz J, et al. Resistance of colon cancer cells to long-term 5-fluorouracil exposure is correlated to the relative level of Bcl-2 and Bcl-X(L) in addition to Bax and p53 status. Int J Cancer. 2002;98:498–504. 11920608 10.1002/ijc.10146

[74] Lavarone E, Barbieri CM, Pasini D. Dissecting the role of H3K27 acetylation and methylation in PRC2 mediated control of cellular identity. Nat Commun. 2019;10:1679. 30976011 10.1038/s41467-019-09624-w PMC6459869

[75] Au SL, Wong CC, Lee JM, Fan DN, Tsang FH, Ng IO, et al. Enhancer of zeste homolog 2 epigenetically silences multiple tumor suppressor microRNAs to promote liver cancer metastasis. Hepatology. 2012;56:622–31. 22370893 10.1002/hep.25679

[76] Bárcena-Varela M, Caruso S, Llerena S, Álvarez-Sola G, Uriarte I, Latasa MU, et al. Dual Targeting of Histone Methyltransferase G9a and DNA-Methyltransferase 1 for the Treatment of Experimental Hepatocellular Carcinoma. Hepatology. 2019;69:587–603. 30014490 10.1002/hep.30168

[77] Chiba T, Saito T, Yuki K, Zen Y, Koide S, Kanogawa N, et al. Histone lysine methyltransferase SUV39H1 is a potent target for epigenetic therapy of hepatocellular carcinoma. Int J Cancer. 2015;136:289–98. 24844570 10.1002/ijc.28985

[78] Wang DY, Zou LP, Liu XJ, Zhu HG, Zhu R. Hepatitis B virus X protein induces the histone H3 lysine 9 trimethylation on the promoter of p16 gene in hepatocarcinogenesis. Exp Mol Pathol. 2015;99:399–408. 26341139 10.1016/j.yexmp.2015.08.020

[79] Dawson MA, Kouzarides T, Huntly BJP. Targeting Epigenetic Readers in Cancer. N. Engl J Med. 2012;367:647–57. 22894577 10.1056/NEJMra1112635

[80] Bayo J, Fiore EJ, Dominguez LM, Real A, Malvicini M, Rizzo M, et al. A comprehensive study of epigenetic alterations in hepatocellular carcinoma identifies potential therapeutic targets. J Hepatol. 2019;71:78–90. 30880225 10.1016/j.jhep.2019.03.007

[81] Fang D, Wang MR, Guan JL, Han YY, Sheng JQ, Tian DA, et al. Bromodomain-containing protein 9 promotes hepatocellular carcinoma progression via activating the Wnt/β-catenin signaling pathway. Exp Cell Res. 2021;406:112727. 34370992 10.1016/j.yexcr.2021.112727

[82] Dou C, Sun L, Wang L, Cheng J, Wu W, Zhang C, et al. Bromodomain-containing protein 9 promotes the growth and metastasis of human hepatocellular carcinoma by activating the TUFT1/AKT pathway. Cell Death Dis. 2020;11:730. 32908135 10.1038/s41419-020-02943-7 PMC7481201

[83] Zou Z, Huang B, Wu X, Zhang H, Qi J, Bradner J, et al. Brd4 maintains constitutively active NF-κB in cancer cells by binding to acetylated RelA. Oncogene. 2014;33:2395–404. 23686307 10.1038/onc.2013.179 PMC3913736

[84] Zhang P, Dong Z, Cai J, Zhang C, Shen Z, Ke A, et al. BRD4 promotes tumor growth and epithelial-mesenchymal transition in hepatocellular carcinoma. Int J Immunopathol Pharm. 2015;28:36–44. 10.1177/0394632015572070 25816404

[85] Splinter E, de Laat W. The complex transcription regulatory landscape of our genome: control in three dimensions. Embo j. 2011;30:4345–55. 21952046 10.1038/emboj.2011.344 PMC3230377

[86] Hadjur S, Williams LM, Ryan NK, Cobb BS, Sexton T, Fraser P, et al. Cohesins form chromosomal cis-interactions at the developmentally regulated IFNG locus. Nature. 2009;460:410–3. 19458616 10.1038/nature08079 PMC2869028

[87] Jeon AJ, Anene-Nzelu CG, Teo YY, Chong SL, Sekar K, Wu L, et al. A genomic enhancer signature associates with hepatocellular carcinoma prognosis. JHEP Rep. 2023;5:100715. 37168287 10.1016/j.jhepr.2023.100715 PMC10165154

[88] Tsai CN, Yu SC, Lee CW, Pang JS, Wu CH, Lin SE, et al. SOX4 activates CXCL12 in hepatocellular carcinoma cells to modulate endothelial cell migration and angiogenesis in vivo. Oncogene. 2020;39:4695–710. 32404985 10.1038/s41388-020-1319-z

[89] Shih T-C, Wang L, Wang H-C, Wan Y-JY. Glypican-3: A molecular marker for the detection and treatment of hepatocellular carcinoma. Liver Res. 2020;4:168–72. 33384879 10.1016/j.livres.2020.11.003 PMC7771890

[90] Gröschel S, Sanders MA, Hoogenboezem R, de Wit E, Bouwman BAM, Erpelinck C, et al. A single oncogenic enhancer rearrangement causes concomitant EVI1 and GATA2 deregulation in leukemia. Cell. 2014;157:369–81. 24703711 10.1016/j.cell.2014.02.019

[91] Dowen JM, Fan ZP, Hnisz D, Ren G, Abraham BJ, Zhang LN, et al. Control of cell identity genes occurs in insulated neighborhoods in mammalian chromosomes. Cell. 2014;159:374–87. 25303531 10.1016/j.cell.2014.09.030 PMC4197132

[92] Mansour MR, Abraham BJ, Anders L, Berezovskaya A, Gutierrez A, Durbin AD, et al. Oncogene regulation. An oncogenic super-enhancer formed through somatic mutation of a noncoding intergenic element. Science. 2014;346:1373–7. 25394790 10.1126/science.1259037 PMC4720521

[93] Tsang FH, Law CT, Tang TC, Cheng CL, Chin DW, Tam WV, et al. Aberrant Super-Enhancer Landscape in Human Hepatocellular Carcinoma. Hepatology. 2019;69:2502–17. 30723918 10.1002/hep.30544

[94] Zhang C, Wei S, Sun WP, Teng K, Dai MM, Wang FW, et al. Super-enhancer-driven AJUBA is activated by TCF4 and involved in epithelial-mesenchymal transition in the progression of Hepatocellular Carcinoma. Theranostics. 2020;10:9066–82. 32802179 10.7150/thno.45349 PMC7415796

[95] Shang X-Y, Shi Y, He D-D, Wang L, Luo Q, Deng C-H, et al. ARID1A deficiency weakens BRG1-RAD21 interaction that jeopardizes chromatin compactness and drives liver cancer cell metastasis. Cell Death Dis. 2021;12:990. 34689165 10.1038/s41419-021-04291-6 PMC8542038

[96] Williams AE. Functional aspects of animal microRNAs. Cell Mol Life Sci. 2008;65:545–62. 17965831 10.1007/s00018-007-7355-9 PMC11131689

[97] Kan RL, Chen J, Sallam T. Crosstalk between epitranscriptomic and epigenetic mechanisms in gene regulation. Trends Genet. 2022;38:182–93. 34294427 10.1016/j.tig.2021.06.014 PMC9093201

[98] Esquela-Kerscher A, Slack FJ. Oncomirs - microRNAs with a role in cancer. Nat Rev Cancer. 2006;6:259–69. 16557279 10.1038/nrc1840

[99] Calin GA, Croce CM. MicroRNA signatures in human cancers. Nat Rev Cancer. 2006;6:857–66. 17060945 10.1038/nrc1997

[100] Roundtree IA, Evans ME, Pan T, He C. Dynamic RNA Modifications in Gene Expression Regulation. Cell. 2017;169:1187–200. 28622506 10.1016/j.cell.2017.05.045 PMC5657247

[101] Cao L-q, Yang X-w, Chen Y-b, Zhang D-w, Jiang X-F, Xue P. Exosomal miR-21 regulates the TETs/PTENp1/PTEN pathway to promote hepatocellular carcinoma growth. Mol Cancer. 2019;18:148. 31656200 10.1186/s12943-019-1075-2 PMC6815431

[102] Fornari F, Gramantieri L, Ferracin M, Veronese A, Sabbioni S, Calin GA, et al. MiR-221 controls CDKN1C/p57 and CDKN1B/p27 expression in human hepatocellular carcinoma. Oncogene. 2008;27:5651–61. 18521080 10.1038/onc.2008.178

[103] Hung J-H, Li C-H, Yeh C-H, Huang P-C, Fang C-C, Chen Y-F, et al. MicroRNA-224 down-regulates Glycine N-methyltransferase gene expression in Hepatocellular Carcinoma. Sci Rep. 2018;8:12284. 30115977 10.1038/s41598-018-30682-5 PMC6095880

[104] Chen Q, Yin D, Zhang Y, Yu L, Li X-D, Zhou Z-J, et al. MicroRNA-29a induces loss of 5-hydroxymethylcytosine and promotes metastasis of hepatocellular carcinoma through a TET–SOCS1–MMP9 signaling axis. Cell Death Dis. 2017;8:e2906. 28661477 10.1038/cddis.2017.142 PMC5520877

[105] Zhang J, Yang Y, Yang T, Liu Y, Li A, Fu S, et al. microRNA-22, downregulated in hepatocellular carcinoma and correlated with prognosis, suppresses cell proliferation and tumourigenicity. Br J Cancer. 2010;103:1215–20. 20842113 10.1038/sj.bjc.6605895 PMC2967065

[106] Furuta M, Kozaki K-i, Tanaka S, Arii S, Imoto I, Inazawa J. miR-124 and miR-203 are epigenetically silenced tumor-suppressive microRNAs in hepatocellular carcinoma. Carcinogenesis. 2009;31:766–76. 19843643 10.1093/carcin/bgp250

[107] Yan X, Zhu Z, Xu S, Yang L-n, Liao X-H, Zheng M, et al. MicroRNA-140-5p inhibits hepatocellular carcinoma by directly targeting the unique isomerase Pin1 to block multiple cancer-driving pathways. Sci Rep. 2017;7:45915. 28383568 10.1038/srep45915 PMC5382892

[108] Zhang S, Shan C, Kong G, Du Y, Ye L, Zhang X. MicroRNA-520e suppresses growth of hepatoma cells by targeting the NF-κB-inducing kinase (NIK). Oncogene. 2012;31:3607–20. 22105365 10.1038/onc.2011.523

[109] Zhao J, Li H, Zhao S, Wang E, Zhu J, Feng D, et al. Epigenetic silencing of miR-144/451a cluster contributes to HCC progression via paracrine HGF/MIF-mediated TAM remodeling. Mol Cancer. 2021;20:46. 33658044 10.1186/s12943-021-01343-5 PMC7927270

[110] Zhang L, Yang F, Yuan J-H, Yuan S-X, Zhou W-P, Huo X-S, et al. Epigenetic activation of the MiR-200 family contributes to H19-mediated metastasis suppression in hepatocellular carcinoma. Carcinogenesis. 2012;34:577–86. 23222811 10.1093/carcin/bgs381

[111] Zhang Y, Wei Y, Li X, Liang X, Wang L, Song J, et al. microRNA-874 suppresses tumor proliferation and metastasis in hepatocellular carcinoma by targeting the DOR/EGFR/ERK pathway. Cell Death Dis. 2018;9:130. 29374140 10.1038/s41419-017-0131-3 PMC5833540

[112] Zhao Z, Meng J, Su R, Zhang J, Chen J, Ma X, et al. Epitranscriptomics in liver disease: Basic concepts and therapeutic potential. J Hepatol. 2020;73:664–79. 32330603 10.1016/j.jhep.2020.04.009

[113] Boriack-Sjodin PA, Ribich S, Copeland RA. RNA-modifying proteins as anticancer drug targets. Nat Rev Drug Discov. 2018;17:435–53. 29773918 10.1038/nrd.2018.71

[114] Ortiz-Barahona V, Soler M, Davalos V, García-Prieto CA, Janin M, Setien F, et al. Epigenetic inactivation of the 5-methylcytosine RNA methyltransferase NSUN7 is associated with clinical outcome and therapeutic vulnerability in liver cancer. Mol Cancer. 2023;22:83. 37173708 10.1186/s12943-023-01785-z PMC10176850

[115] Chen M, Wei L, Law CT, Tsang FH, Shen J, Cheng CL, et al. RNA N6-methyladenosine methyltransferase-like 3 promotes liver cancer progression through YTHDF2-dependent posttranscriptional silencing of SOCS2. Hepatology. 2018;67:2254–70. 29171881 10.1002/hep.29683

[116] Villanueva A. Hepatocellular Carcinoma. N. Engl J Med. 2019;380:1450–62. 30970190 10.1056/NEJMra1713263

[117] Llovet JM, Willoughby CE, Singal AG, Greten TF, Heikenwälder M, El-Serag HB, et al. Nonalcoholic steatohepatitis-related hepatocellular carcinoma: pathogenesis and treatment. Nat Rev Gastroenterol Hepatol. 2023;20:487–503. 36932227 10.1038/s41575-023-00754-7 PMC12165718

[118] Cleary SP, Jeck WR, Zhao X, Chen K, Selitsky SR, Savich GL, et al. Identification of driver genes in hepatocellular carcinoma by exome sequencing. Hepatology. 2013;58:1693–702. 23728943 10.1002/hep.26540 PMC3830584

[119] Lee J, Kim DH, Lee S, Yang QH, Lee DK, Lee SK, et al. A tumor suppressive coactivator complex of p53 containing ASC-2 and histone H3-lysine-4 methyltransferase MLL3 or its paralogue MLL4. Proc Natl Acad Sci USA. 2009;106:8513–8. 19433796 10.1073/pnas.0902873106 PMC2689008

[120] Inoue Y, Suzuki Y, Kunishima Y, Washio T, Morishita S, Takeda H. High-fat diet in early life triggers both reversible and persistent epigenetic changes in the medaka fish (Oryzias latipes). BMC Genomics. 2023;24:472. 37605229 10.1186/s12864-023-09557-1 PMC10441761

[121] Reddy MA, Zhang E, Natarajan R. Epigenetic mechanisms in diabetic complications and metabolic memory. Diabetologia. 2015;58:443–55. 25481708 10.1007/s00125-014-3462-y PMC4324095

[122] Dong H, Sun Y, Nie L, Cui A, Zhao P, Leung WK, et al. Metabolic memory: mechanisms and diseases. Signal Transduct Target Ther. 2024;9:38. 38413567 10.1038/s41392-024-01755-x PMC10899265

[123] Zeybel M, Hardy T, Robinson SM, Fox C, Anstee QM, Ness T, et al. Differential DNA methylation of genes involved in fibrosis progression in non-alcoholic fatty liver disease and alcoholic liver disease. Clin Epigenetics. 2015;7:25. 25859289 10.1186/s13148-015-0056-6 PMC4391139

[124] Lai Z, Chen J, Ding C, Wong K, Chen X, Pu L, et al. Association of Hepatic Global DNA Methylation and Serum One-Carbon Metabolites with Histological Severity in Patients with NAFLD. Obesity. 2020;28:197–205. 31785086 10.1002/oby.22667

[125] Herranz JM, López-Pascual A, Clavería-Cabello A, Uriarte I, Latasa MU, Irigaray-Miramon A, et al. Comprehensive analysis of epigenetic and epitranscriptomic genes’ expression in human NAFLD. J Physiol Biochem. 2023;79:901–24. 37620598 10.1007/s13105-023-00976-y PMC10636027

[126] Jühling F, Hamdane N, Crouchet E, Li S, El Saghire H, Mukherji A, et al. Targeting clinical epigenetic reprogramming for chemoprevention of metabolic and viral hepatocellular carcinoma. Gut. 2021;70:157–69. 32217639 10.1136/gutjnl-2019-318918 PMC7116473

[127] Pfister D, Núñez NG, Pinyol R, Govaere O, Pinter M, Szydlowska M, et al. NASH limits anti-tumour surveillance in immunotherapy-treated HCC. Nature. 2021;592:450–6. 33762733 10.1038/s41586-021-03362-0 PMC8046670

[128] Fujisawa T, Filippakopoulos P. Functions of bromodomain-containing proteins and their roles in homeostasis and cancer. Nat Rev Mol Cell Biol. 2017;18:246–62. 28053347 10.1038/nrm.2016.143

[129] Zhang W, Chen J, Wu M, Zhang X, Zhang M, Yue L, et al. PRMT5 restricts hepatitis B virus replication through epigenetic repression of covalently closed circular DNA transcription and interference with pregenomic RNA encapsidation. Hepatology. 2017;66:398–415. 28236308 10.1002/hep.29133

[130] Anstee QM, Reeves HL, Kotsiliti E, Govaere O, Heikenwalder M. From NASH to HCC: current concepts and future challenges. Nat Rev Gastroenterol Hepatol. 2019;16:411–28. 31028350 10.1038/s41575-019-0145-7

[131] Johnson P, Zhou Q, Dao DY, Lo YMD. Circulating biomarkers in the diagnosis and management of hepatocellular carcinoma. Nat Rev Gastroenterol Hepatol. 2022;19:670–81. 35676420 10.1038/s41575-022-00620-y

[132] Ye Q, Ling S, Zheng S, Xu X. Liquid biopsy in hepatocellular carcinoma: circulating tumor cells and circulating tumor DNA. Mol Cancer. 2019;18:114. 31269959 10.1186/s12943-019-1043-x PMC6607541

[133] Rashid S, Sun Y, Ali Khan Saddozai U, Hayyat S, Munir MU, Akbar MU, et al. Circulating tumor DNA and its role in detection, prognosis and therapeutics of hepatocellular carcinoma. Chin J Cancer Res. 2024;36:195–214. 38751441 10.21147/j.issn.1000-9604.2024.02.07 PMC11090798

[134] Tischoff I, Tannapfe A. DNA methylation in hepatocellular carcinoma. World J Gastroenterol. 2008;14:1741–8. 18350605 10.3748/wjg.14.1741 PMC2695914

[135] Xu RH, Wei W, Krawczyk M, Wang W, Luo H, Flagg K, et al. Circulating tumour DNA methylation markers for diagnosis and prognosis of hepatocellular carcinoma. Nat Mater. 2017;16:1155–61. 29035356 10.1038/nmat4997

[136] Deng Z, Ji Y, Han B, Tan Z, Ren Y, Gao J, et al. Early detection of hepatocellular carcinoma via no end-repair enzymatic methylation sequencing of cell-free DNA and pre-trained neural network. Genome Med. 2023;15:93. 37936230 10.1186/s13073-023-01238-8 PMC10631027

[137] Kim SC, Kim DW, Cho EJ, Lee JY, Kim J, Kwon C, et al. A circulating cell-free DNA methylation signature for the detection of hepatocellular carcinoma. Mol Cancer. 2023;22:164. 37803338 10.1186/s12943-023-01872-1 PMC10557228

[138] Füllgrabe J, Gosal WS, Creed P, Liu S, Lumby CK, Morley DJ, et al. Simultaneous sequencing of genetic and epigenetic bases in DNA. Nat Biotechnol. 2023;41:1457–64. 36747096 10.1038/s41587-022-01652-0 PMC10567558

[139] Cai Z, Zhang J, He Y, Xia L, Dong X, Chen G, et al. Liquid biopsy by combining 5-hydroxymethylcytosine signatures of plasma cell-free DNA and protein biomarkers for diagnosis and prognosis of hepatocellular carcinoma. ESMO Open. 2021;6:100021. 33508734 10.1016/j.esmoop.2020.100021 PMC7841321

[140] Zhu Q, Xie J, Mei W, Zeng C. Methylated circulating tumor DNA in hepatocellular carcinoma: A comprehensive analysis of biomarker potential and clinical implications. Cancer Treat Rev. 2024;128:102763. 38763055 10.1016/j.ctrv.2024.102763

[141] Guo DZ, Huang A, Wang YC, Zhou S, Wang H, Xing XL, et al. Early detection and prognosis evaluation for hepatocellular carcinoma by circulating tumour DNA methylation: A multicentre cohort study. Clin Transl Med. 2024;14:e1652. 38741204 10.1002/ctm2.1652 PMC11091019

[142] Fu S, Debes JD, Boonstra A. DNA methylation markers in the detection of hepatocellular carcinoma. Eur J Cancer. 2023;191:112960.10.1016/j.ejca.2023.11296037473464

[143] Lin X-J, Chong Y, Guo Z-W, Xie C, Yang X-J, Zhang Q, et al. A serum microRNA classifier for early detection of hepatocellular carcinoma: a multicentre, retrospective, longitudinal biomarker identification study with a nested case-control study. Lancet Oncol. 2015;16:804–15. 26088272 10.1016/S1470-2045(15)00048-0

[144] Jin Y, Wong YS, Goh BKP, Chan CY, Cheow PC, Chow PKH, et al. Circulating microRNAs as Potential Diagnostic and Prognostic Biomarkers in Hepatocellular Carcinoma. Sci Rep. 2019;9:10464. 31320713 10.1038/s41598-019-46872-8 PMC6639394

[145] Yerukala Sathipati S, Ho S-Y. Novel miRNA signature for predicting the stage of hepatocellular carcinoma. Sci Rep. 2020;10:14452. 32879391 10.1038/s41598-020-71324-z PMC7467934

[146] Fornari F, Pollutri D, Patrizi C, La Bella T, Marinelli S, Casadei Gardini A, et al. In Hepatocellular Carcinoma miR-221 Modulates Sorafenib Resistance through Inhibition of Caspase-3–Mediated Apoptosis. Clin Cancer Res. 2017;23:3953–65. 28096271 10.1158/1078-0432.CCR-16-1464

[147] Xia H, Ooi LL, Hui KM. MicroRNA-216a/217-induced epithelial-mesenchymal transition targets PTEN and SMAD7 to promote drug resistance and recurrence of liver cancer. Hepatology. 2013;58:629–41. 23471579 10.1002/hep.26369

[148] Fernández-Tussy P, Rodríguez-Agudo R, Fernández-Ramos D, Barbier-Torres L, Zubiete-Franco I, SLd Davalillo, et al. Anti-miR-518d-5p overcomes liver tumor cell death resistance through mitochondrial activity. Cell Death Dis. 2021;12:555. 34050139 10.1038/s41419-021-03827-0 PMC8163806

[149] Dhanasekaran R, Nault JC, Roberts LR, Zucman-Rossi J. Genomic Medicine and Implications for Hepatocellular Carcinoma Prevention and Therapy. Gastroenterology. 2019;156:492–509. 30404026 10.1053/j.gastro.2018.11.001 PMC6340723

[150] Turcios VilchezV, Marti L, Gedaly F. R. Targeting Wnt/β-catenin pathway in hepatocellular carcinoma treatment. World J Gastroenterol. 2016;22:823–32. 26811628 10.3748/wjg.v22.i2.823 PMC4716080

[151] Hussain SP, Schwank J, Staib F, Wang XW, Harris CC. TP53 mutations and hepatocellular carcinoma: insights into the etiology and pathogenesis of liver cancer. Oncogene. 2007;26:2166–76. 17401425 10.1038/sj.onc.1210279

[152] Li W, Yan Y, Zheng Z, Zhu Q, Long Q, Sui S, et al. Targeting the NCOA3-SP1-TERT axis for tumor growth in hepatocellular carcinoma. Cell Death Dis. 2020;11:1011. 33239622 10.1038/s41419-020-03218-x PMC7689448

[153] Faivre S, Rimassa L, Finn RS. Molecular therapies for HCC: Looking outside the box. J Hepatol. 2020;72:342–52. 31954496 10.1016/j.jhep.2019.09.010

[154] Kornblith AB, Herndon JE 2nd, Silverman LR, Demakos EP, Odchimar-Reissig R, et al. Impact of azacytidine on the quality of life of patients with myelodysplastic syndrome treated in a randomized phase III trial: a Cancer and Leukemia Group B study. J Clin Oncol. 2002;20:2441–52. 12011121 10.1200/JCO.2002.04.044

[155] Mann BS, Johnson JR, Cohen MH, Justice R, Pazdur R. FDA approval summary: vorinostat for treatment of advanced primary cutaneous T-cell lymphoma. Oncologist. 2007;12:1247–52. 17962618 10.1634/theoncologist.12-10-1247

[156] Lee HZ, Kwitkowski VE, Del Valle PL, Ricci MS, Saber H, Habtemariam BA, et al. FDA Approval: Belinostat for the Treatment of Patients with Relapsed or Refractory Peripheral T-cell Lymphoma. Clin Cancer Res. 2015;21:2666–70. 25802282 10.1158/1078-0432.CCR-14-3119

[157] Mei Q, Chen M, Lu X, Li X, Duan F, Wang M, et al. An open-label, single-arm, phase I/II study of lower-dose decitabine based therapy in patients with advanced hepatocellular carcinoma. Oncotarget. 2015;6:16698–711. 25895027 10.18632/oncotarget.3677 PMC4599300

[158] Bitzer M, Horger M, Giannini EG, Ganten TM, Wörns MA, Siveke JT, et al. Resminostat plus sorafenib as second-line therapy of advanced hepatocellular carcinoma - The SHELTER study. J Hepatol. 2016;65:280–8. 26952006 10.1016/j.jhep.2016.02.043

[159] Liu YC, Su CW, Ko PS, Lee RC, Liu CJ, Huang YH, et al. A clinical trial with valproic acid and hydralazine in combination with gemcitabine and cisplatin followed by doxorubicin and dacarbazine for advanced hepatocellular carcinoma. Asia Pac J Clin Oncol. 2022;18:19–27. 32964588 10.1111/ajco.13443

[160] Gnyszka A, Jastrzębski Z, Flis S. DNA Methyltransferase Inhibitors and Their Emerging Role in Epigenetic Therapy of Cancer. Anticancer Res. 2013;33:2989–96. 23898051

[161] Jueliger S, Lyons J, Cannito S, Pata I, Pata P, Shkolnaya M, et al. Efficacy and epigenetic interactions of novel DNA hypomethylating agent guadecitabine (SGI-110) in preclinical models of hepatocellular carcinoma. Epigenetics. 2016;11:709–20. 27646854 10.1080/15592294.2016.1214781 PMC5094635

[162] Gailhouste L, Liew LC, Yasukawa K, Hatada I, Tanaka Y, Nakagama H, et al. Differentiation Therapy by Epigenetic Reconditioning Exerts Antitumor Effects on Liver Cancer Cells. Mol Ther. 2018;26:1840–54. 29759938 10.1016/j.ymthe.2018.04.018 PMC6035736

[163] Kuang Y, El-Khoueiry A, Taverna P, Ljungman M, Neamati N. Guadecitabine (SGI-110) priming sensitizes hepatocellular carcinoma cells to oxaliplatin. Mol Oncol. 2015;9:1799–814. 26160429 10.1016/j.molonc.2015.06.002 PMC5528722

[164] Liu M, Zhang L, Li H, Hinoue T, Zhou W, Ohtani H, et al. Integrative Epigenetic Analysis Reveals Therapeutic Targets to the DNA Methyltransferase Inhibitor Guadecitabine (SGI-110) in Hepatocellular Carcinoma. Hepatology. 2018;68:1412–28. 29774579 10.1002/hep.30091 PMC6173644

[165] Seto E, Yoshida M. Erasers of histone acetylation: the histone deacetylase enzymes. Cold Spring Harb Perspect Biol. 2014;6:a018713. 24691964 10.1101/cshperspect.a018713 PMC3970420

[166] Fu M, Shi W, Li Z, Liu H. Activation of mPTP-dependent mitochondrial apoptosis pathway by a novel pan HDAC inhibitor resminostat in hepatocellular carcinoma cells. Biochem Biophys Res Commun. 2016;477:527–33. 27144317 10.1016/j.bbrc.2016.04.147

[167] Streubel G, Schrepfer S, Kallus H, Parnitzke U, Wulff T, Hermann F, et al. Histone deacetylase inhibitor resminostat in combination with sorafenib counteracts platelet-mediated pro-tumoral effects in hepatocellular carcinoma. Sci Rep. 2021;11:9587. 33953226 10.1038/s41598-021-88983-1 PMC8100298

[168] Di Fazio P, Schneider-Stock R, Neureiter D, Okamoto K, Wissniowski T, Gahr S, et al. The pan-deacetylase inhibitor panobinostat inhibits growth of hepatocellular carcinoma models by alternative pathways of apoptosis. Cell Oncol. 2010;32:285–300. 20208142 10.3233/CLO-2010-0511 PMC4619232

[169] Lachenmayer A, Toffanin S, Cabellos L, Alsinet C, Hoshida Y, Villanueva A, et al. Combination therapy for hepatocellular carcinoma: additive preclinical efficacy of the HDAC inhibitor panobinostat with sorafenib. J Hepatol. 2012;56:1343–50. 22322234 10.1016/j.jhep.2012.01.009 PMC3355195

[170] Kunnimalaiyaan S, Sokolowski K, Gamblin TC, Kunnimalaiyaan M. Suberoylanilide hydroxamic Acid, a histone deacetylase inhibitor, alters multiple signaling pathways in hepatocellular carcinoma cell lines. Am J Surg. 2017;213:645–51. 28007318 10.1016/j.amjsurg.2016.12.001

[171] Yuan H, Li AJ, Ma SL, Cui LJ, Wu B, Yin L, et al. Inhibition of autophagy significantly enhances combination therapy with sorafenib and HDAC inhibitors for human hepatoma cells. World J Gastroenterol. 2014;20:4953–62. 24833845 10.3748/wjg.v20.i17.4953 PMC4009527

[172] Machado MC, Bellodi-Privato M, Kubrusly MS, Molan NA, Tharcisio T Jr, de Oliveira ER, et al. Valproic acid inhibits human hepatocellular cancer cells growth in vitro and in vivo. J Exp Ther Oncol. 2011;9:85–92. 21699015

[173] Liu J, Yang X, Liang Q, Yu Y, Shen X, Sun G. Valproic acid overcomes sorafenib resistance by reducing the migration of Jagged2-mediated Notch1 signaling pathway in hepatocellular carcinoma cells. Int J Biochem Cell Biol. 2020;126:105820. 32750425 10.1016/j.biocel.2020.105820

[174] Wang H, Guo Y, Fu M, Liang X, Zhang X, Wang R, et al. Antitumor activity of Chidamide in hepatocellular carcinoma cell lines. Mol Med Rep. 2012;5:1503–8. 22484326 10.3892/mmr.2012.858

[175] Sun WJ, Huang H, He B, Hu DH, Li PH, Yu YJ, et al. Romidepsin induces G2/M phase arrest via Erk/cdc25C/cdc2/cyclinB pathway and apoptosis induction through JNK/c-Jun/caspase3 pathway in hepatocellular carcinoma cells. Biochem Pharm. 2017;127:90–100. 28012958 10.1016/j.bcp.2016.12.008

[176] Eyre TA, Collins GP, Gupta A, Coupe N, Sheikh S, Whittaker J, et al. A phase 1 study to assess the safety, tolerability, and pharmacokinetics of CXD101 in patients with advanced cancer. Cancer. 2019;125:99–108. 30332497 10.1002/cncr.31791

[177] Straining R, Eighmy W. Tazemetostat: EZH2 Inhibitor. J Adv Pr Oncol. 2022;13:158–63. 10.6004/jadpro.2022.13.2.7 PMC8955562 35369397

[178] Amin MN, El-Far YM, El-Mowafy M, Elgaml A. Tazemetostat decreases β-catenin and CD13 protein expression in HEPG-2 and Hepatitis B virus-transfected HEPG-2 with decreased cell viability. Clin Epigenetics. 2023;15:180. 37941056 10.1186/s13148-023-01593-8 PMC10634085

[179] Stein EM, Garcia-Manero G, Rizzieri DA, Tibes R, Berdeja JG, Savona MR, et al. The DOT1L inhibitor pinometostat reduces H3K79 methylation and has modest clinical activity in adult acute leukemia. Blood. 2018;131:2661–9. 29724899 10.1182/blood-2017-12-818948 PMC6265654

[180] Yang YB, Wu CY, Wang XY, Deng J, Cao WJ, Tang YZ, et al. Targeting inflammatory macrophages rebuilds therapeutic efficacy of DOT1L inhibition in hepatocellular carcinoma. Mol Ther. 2023;31:105–18. 36183166 10.1016/j.ymthe.2022.09.019 PMC9840147

[181] Liu TP, Lo HL, Wei LS, Hsiao HH, Yang PM. S-Adenosyl-L-methionine-competitive inhibitors of the histone methyltransferase EZH2 induce autophagy and enhance drug sensitivity in cancer cells. Anticancer Drugs. 2015;26:139–47. 25203626 10.1097/CAD.0000000000000166 PMC4276571

[182] Sang N, Zhong X, Gou K, Liu H, Xu J, Zhou Y, et al. Pharmacological inhibition of LSD1 suppresses growth of hepatocellular carcinoma by inducing GADD45B. MedComm. 2023;4:e269. (2020) 37250145 10.1002/mco2.269 PMC10209615

[183] Lee DH, Kim GW, Jeon YH, Yoo J, Lee SW, Kwon SH. Advances in histone demethylase KDM4 as cancer therapeutic targets. Faseb J. 2020;34:3461–84. 31961018 10.1096/fj.201902584R

[184] Kim SY, Hwang S, Lee BR, Hong JA, Sung YH, Kim I. Inhibition of histone demethylase KDM4 by ML324 induces apoptosis through the unfolded protein response and Bim upregulation in hepatocellular carcinoma cells. Chem Biol Interact. 2022;353:109806. 34999051 10.1016/j.cbi.2022.109806

[185] Kantidze OL, Luzhin AV, Nizovtseva EV, Safina A, Valieva ME, Golov AK, et al. The anti-cancer drugs curaxins target spatial genome organization. Nat Commun. 2019;10:1441. 30926878 10.1038/s41467-019-09500-7 PMC6441033

[186] Garcia H, Miecznikowski JC, Safina A, Commane M, Ruusulehto A, Kilpinen S, et al. Facilitates chromatin transcription complex is an “accelerator” of tumor transformation and potential marker and target of aggressive cancers. Cell Rep. 2013;4:159–73. 23831030 10.1016/j.celrep.2013.06.013 PMC5886782

[187] Gasparian AV, Burkhart CA, Purmal AA, Brodsky L, Pal M, Saranadasa M, et al. Curaxins: anticancer compounds that simultaneously suppress NF-κB and activate p53 by targeting FACT. Sci Transl Med. 2011;3:95ra74. 21832239 10.1126/scitranslmed.3002530 PMC6281439

[188] Shen J, Chen M, Lee D, Law CT, Wei L, Tsang FH, et al. Histone chaperone FACT complex mediates oxidative stress response to promote liver cancer progression. Gut. 2020;69:329–42. 31439637 10.1136/gutjnl-2019-318668

[189] Callegari E, Elamin BK, Giannone F, Milazzo M, Altavilla G, Fornari F, et al. Liver tumorigenicity promoted by microRNA-221 in a mouse transgenic model. Hepatology. 2012;56:1025–33. 22473819 10.1002/hep.25747

[190] Tsai WC, Hsu PW, Lai TC, Chau GY, Lin CW, Chen CM, et al. MicroRNA-122, a tumor suppressor microRNA that regulates intrahepatic metastasis of hepatocellular carcinoma. Hepatology. 2009;49:1571–82. 19296470 10.1002/hep.22806

[191] van der Ree MH, de Vree JM, Stelma F, Willemse S, van der Valk M, Rietdijk S, et al. Safety, tolerability, and antiviral effect of RG-101 in patients with chronic hepatitis C: a phase 1B, double-blind, randomised controlled trial. Lancet. 2017;389:709–17. 28087069 10.1016/S0140-6736(16)31715-9

[192] Wei SC, Duffy CR, Allison JP. Fundamental Mechanisms of Immune Checkpoint Blockade Therapy. Cancer Discov. 2018;8:1069–86. 30115704 10.1158/2159-8290.CD-18-0367

[193] Villanueva L, Álvarez-Errico D, Esteller M. The Contribution of Epigenetics to Cancer Immunotherapy. Trends Immunol. 2020;41:676–91. 32622854 10.1016/j.it.2020.06.002

[194] Akce M, El-Rayes BF, Wajapeyee N. Combinatorial targeting of immune checkpoints and epigenetic regulators for hepatocellular carcinoma therapy. Oncogene. 2023;42:1051–7. 36854723 10.1038/s41388-023-02646-1

[195] Yang J, Xu J, Wang W, Zhang B, Yu X, Shi S. Epigenetic regulation in the tumor microenvironment: molecular mechanisms and therapeutic targets. Signal Transduct Target Ther. 2023;8:210. 37217462 10.1038/s41392-023-01480-x PMC10203321

[196] Capece D, Fischietti M, Verzella D, Gaggiano A, Cicciarelli G, Tessitore A, et al. The Inflammatory Microenvironment in Hepatocellular Carcinoma: A Pivotal Role for Tumor-Associated Macrophages. BioMed Res Int. 2013;2013:187204. 23533994 10.1155/2013/187204 PMC3591180

[197] Qiu W, Wang B, Gao Y, Tian Y, Tian M, Chen Y, et al. Targeting Histone Deacetylase 6 Reprograms Interleukin-17-Producing Helper T Cell Pathogenicity and Facilitates Immunotherapies for Hepatocellular Carcinoma. Hepatology. 2020;71:1967–87. 31539182 10.1002/hep.30960

[198] Tu Y, Wu H, Zhong C, Liu Y, Xiong Z, Chen S, et al. Pharmacological activation of STAT1-GSDME pyroptotic circuitry reinforces epigenetic immunotherapy for hepatocellular carcinoma. Gut. 2024. 10.1136/gutjnl-2024-332281. Epub ahead of print. 10.1136/gutjnl-2024-332281 PMC12013592 39486886

[199] Wang Y, Cao K. KDM1A Promotes Immunosuppression in Hepatocellular Carcinoma by Regulating PD-L1 through Demethylating MEF2D. J Immunol Res. 2021;2021:9965099. 34307695 10.1155/2021/9965099 PMC8270703

[200] Wang D, Quiros J, Mahuron K, Pai CC, Ranzani V, Young A, et al. Targeting EZH2 Reprograms Intratumoral Regulatory T Cells to Enhance Cancer Immunity. Cell Rep. 2018;23:3262–74. 29898397 10.1016/j.celrep.2018.05.050 PMC6094952

[201] Bugide S, Green MR, Wajapeyee N. Inhibition of Enhancer of zeste homolog 2 (EZH2) induces natural killer cell-mediated eradication of hepatocellular carcinoma cells. Proc Natl Acad Sci USA. 2018;115:E3509–e18. 29581297 10.1073/pnas.1802691115 PMC5899497

[202] Chakravarthy A, Furness A, Joshi K, Ghorani E, Ford K, Ward MJ, et al. Pan-cancer deconvolution of tumour composition using DNA methylation. Nat Commun. 2018;9:3220. 30104673 10.1038/s41467-018-05570-1 PMC6089972

[203] Peng D, Kryczek I, Nagarsheth N, Zhao L, Wei S, Wang W, et al. Epigenetic silencing of TH1-type chemokines shapes tumour immunity and immunotherapy. Nature. 2015;527:249–53. 26503055 10.1038/nature15520 PMC4779053

[204] Xiao G, Jin LL, Liu CQ, Wang YC, Meng YM, Zhou ZG, et al. EZH2 negatively regulates PD-L1 expression in hepatocellular carcinoma. J Immunother Cancer. 2019;7:300. 31727135 10.1186/s40425-019-0784-9 PMC6854886

[205] Topalian SL, Hodi FS, Brahmer JR, Gettinger SN, Smith DC, McDermott DF, et al. Safety, Activity, and Immune Correlates of Anti–PD-1 Antibody in Cancer. N. Engl J Med. 2012;366:2443–54. 22658127 10.1056/NEJMoa1200690 PMC3544539

[206] Wang M, Yu L, Wei X, Wei Y. Role of tumor gene mutations in treatment response to immune checkpoint blockades. Precis Clin Med. 2019;2:100–9. 35692451 10.1093/pcmedi/pbz006 PMC8985804

[207] Yin Y, Feng W, Chen J, Chen X, Wang G, Wang S, et al. Immunosuppressive tumor microenvironment in the progression, metastasis, and therapy of hepatocellular carcinoma: from bench to bedside. Exp Hematol Oncol. 2024;13:72. 39085965 10.1186/s40164-024-00539-x PMC11292955

[208] Wu R, Guo W, Qiu X, Wang S, Sui C, Lian Q, et al. Comprehensive analysis of spatial architecture in primary liver cancer. Sci Adv. 2021;7:eabg3750. 34919432 10.1126/sciadv.abg3750 PMC8683021

[209] Liu L, Toung JM, Jassowicz AF, Vijayaraghavan R, Kang H, Zhang R, et al. Targeted methylation sequencing of plasma cell-free DNA for cancer detection and classification. Ann Oncol. 2018;29:1445–53. 29635542 10.1093/annonc/mdy119 PMC6005020

[210] Qi LN, Xiang BD, Wu FX, Ye JZ, Zhong JH, Wang YY, et al. Circulating Tumor Cells Undergoing EMT Provide a Metric for Diagnosis and Prognosis of Patients with Hepatocellular Carcinoma. Cancer Res. 2018;78:4731–44. 29915159 10.1158/0008-5472.CAN-17-2459

[211] Sefrioui D, Verdier V, Savoye-Collet C, Beaussire L, Ghomadi S, Gangloff A, et al. Circulating DNA changes are predictive of disease progression after transarterial chemoembolization. Int J Cancer. 2022;150:532–41. 34622951 10.1002/ijc.33829

[212] Shuen TWH, Alunni-Fabbroni M, Öcal E, Malfertheiner P, Wildgruber M, Schinner R, et al. Extracellular Vesicles May Predict Response to Radioembolization and Sorafenib Treatment in Advanced Hepatocellular Carcinoma: An Exploratory Analysis from the SORAMIC Trial. Clin Cancer Res. 2022;28:3890–901. 35763041 10.1158/1078-0432.CCR-22-0569 PMC9433961

[213] Chan Y-T, Zhang C, Wu J, Lu P, Xu L, Yuan H, et al. Biomarkers for diagnosis and therapeutic options in hepatocellular carcinoma. Mol Cancer. 2024;23:189. 39242496 10.1186/s12943-024-02101-z PMC11378508

[214] Le L, Qipeng W, Chunmeng M, Hasnat M, Luyong Z, Zhenzhou J, et al. 5-Azacytidine promotes HCC cell metastasis by up-regulating RDH16 expression. Eur J Pharm. 2023;950:175736. 10.1016/j.ejphar.2023.175736 37116561

[215] Altekruse SF, McGlynn KA, Reichman ME. Hepatocellular carcinoma incidence, mortality, and survival trends in the United States from 1975 to 2005. J Clin Oncol. 2009;27:1485–91. 19224838 10.1200/JCO.2008.20.7753 PMC2668555

[216] Huang R, Wu Y, Zou Z. Combining EZH2 inhibitors with other therapies for solid tumors: more choices for better effects. Epigenomics. 2022;14:1449–64. 36601794 10.2217/epi-2022-0320

